# Drug Repurposing to Identify a Synergistic High-Order Drug Combination to Treat Sunitinib-Resistant Renal Cell Carcinoma

**DOI:** 10.3390/cancers13163978

**Published:** 2021-08-06

**Authors:** Magdalena Rausch, Adriano Rutz, Pierre-Marie Allard, Céline Delucinge-Vivier, Mylène Docquier, Olivier Dormond, Paul J. Dyson, Jean-Luc Wolfender, Patrycja Nowak-Sliwinska

**Affiliations:** 1 School of Pharmaceutical Sciences, University of Geneva, CMU-Rue Michel-Servet 1, CH-1211 Geneva, Switzerland; Magdalena.Rausch@unige.ch (M.R.); Adriano.Rutz@unige.ch (A.R.); pierre-marie.allard@unige.ch (P.-M.A.); jean-luc.wolfender@unige.ch (J.-L.W.); 2 Institute of Pharmaceutical Sciences of Western Switzerland, University of Geneva, CMU-Rue Michel-Servet 1, CH-1211 Geneva, Switzerland; 3 Translational Research Center in Oncohaematology, CH-1211 Geneva, Switzerland; 4 GE3 Genomics Platform, University of Geneva, CH-1211 Geneva, Switzerland; Celine.Delucinge@unige.ch (C.D.-V.); Mylene.Docquier@unige.ch (M.D.); 5 Department of Genetics and Evolution, University of Geneva, CH-1211 Geneva, Switzerland; 6 Department of Visceral Surgery, Lausanne University Hospital and University of Lausanne, 1015 Lausanne, Switzerland; olivier.dormond@chuv.ch; 7 Institute of Chemical Sciences and Engineering, Ecole Polytechnique Fédérale de Lausanne (EPFL), 1015 Lausanne, Switzerland; paul.dyson@epfl.ch

**Keywords:** drug combination, drug-drug interaction, drug repurposing, metabolism, metformin, Rapta-C, synergistic

## Abstract

**Simple Summary:**

In this study, drug combination screening was used to design a multidrug combination consisting of repurposed drugs to treat sunitinib-resistant clear cell renal cell carcinoma. In the frame of this project, the multidrug combination has been optimized and validated and an insight into the mechanism of action is given. The multidrug combinations significantly altered the transcription of genes related to apoptosis and metabolic pathways. Further analysis of the metabolism revealed strong upregulation of the presence of sphingolipids after multidrug combination treatment. Final evaluation for translation of the multidrug combination in ex vivo organoid-like cultures demonstrated significant anti-cancer efficacy.

**Abstract:**

Repurposed drugs have been evaluated for the management of clear cell renal cell carcinoma (ccRCC), but only a few have influenced the overall survival of patients with advanced disease. To combine repurposed non-oncology with oncological drugs, we applied our validated phenotypic method, which consisted of a reduced experimental part and data modeling. A synergistic optimized multidrug combination (ODC) was identified to significantly reduce the energy levels in cancer remaining inactive in non-cancerous cells. The ODC consisted of Rapta-C, erlotinib, metformin and parthenolide and low doses. Molecular and functional analysis of ODC revealed a loss of adhesiveness and induction of apoptosis. Gene-expression network analysis displayed significant alterations in the cellular metabolism, confirmed by LC-MS based metabolomic analysis, highlighting significant changes in the lipid classes. We used heterotypic in vitro 3D co-cultures and ex vivo organoids to validate the activity of the ODC, maintaining an efficacy of over 70%. Our results show that repurposed drugs can be combined to target cancer cells selectively with prominent activity. The strong impact on cell adherence and metabolism indicates a favorable mechanism of action of the ODC to treat ccRCC.

## 1. Introduction

Given the elevated costs of standard drug discovery, followed by a high attrition rate of the drug candidates [1], new strategies have evolved to reposition or repurpose available drugs [2,3]. Drug repositioning and repurposing are commonly used as synonyms [4], referring to identifying new medical indications for approved or investigational drugs. In oncology, drug repositioning usually refers to the use of an anti-cancer drug approved to treat a specific cancer type [5] for the treatment of another cancer type. Importantly, for these drugs, the mechanism of action, the safety profile, and the potential simultaneous intake with other medications for a particular illness is known, further encouraging the rapid approval in the treatment of other disorders, e.g., cancer [6,7].

In the case of difficult-to-treat and treatment-resistant cancer types, e.g., glioblastoma, colorectal carcinoma or clear cell renal cell carcinoma (ccRCC), efficacious treatments are rare and expensive. Existing treatments for ccRCC include targeted agents, i.e., sunitinib, pazopanib or cabozantinib [8]. These drugs bind to tyrosine kinase receptors or molecules downstream in the cell signaling pathway. The doses applied are high, leading to enhanced off-target effects and the appearance of severe side effects, with cancer cells rapidly acquiring resistance to sustain the treatment.

ccRCC is highly infiltrated with immune cells. In 2015, the FDA approved nivolumab [9], an immune checkpoint inhibitor, for the treatment of ccRCC. The approval of nivolumab and ipilimumab [10] in combination followed. In parallel, other immunotherapeutics, pembrolizumab or avelumab, with axitinib (vascular endothelial growth factor receptor (VEGFR) inhibitor) [11] have been approved. These immunotherapies efficiently reduce tumor growth and prolong the overall survival rate of patients with early and advanced staged tumors. The treatment response, however, remains very heterogeneous, and only a small portion of patients benefit from these treatment regimens.

Drug repurposing might offer new therapeutic options and potential combinations with innovative or existing oncological medicines [3,4,6,7,12,13,14,15]. Repurposing of initially non-oncology drugs appears to be beneficial for the treatment of ccRCC. The Repurposing Drugs in Oncology (ReDo) Project was launched to collect data on non-cancer drugs evidenced to have an anti-cancer activity to further develop a database and support new clinical trials [14,16].

Out of eight predicted candidate drugs, pentamidine, an antimicrobial drug used to prevent pneumonia, exhibited anti-cancer activity in vitro and in vivo and proved to be a validated treatment agent for metastatic ccRCC. Artesunate is an anti-malarial drug with anti-cancer, anti-metastatic, and anti-angiogenic activity in cellular and murine xenograft models [17]. Alternatively, gene expression profiling was used to discover drug candidates repurposed for the treatment of ccRCC. Gene expression signatures of individual tumor samples have been used to personalize drug repurposing [18]. Koudijs et al. collected tumor (*n* = 534) and matched normal (*n* = 72) samples from 530 ccRCC patients to create tumor signatures to connect these to drug signatures [19]. The unique ccRCC gene expression signatures revealed drug candidates for repositioning, with a strong tendency to target small-molecule-based drugs, with erlotinib (epidermal growth factor receptor (EGFR) inhibitor) being one of the top eight drug candidates.

Currently, most studies applying repurposed drugs for the treatment of ccRCC describe in vitro and in vivo results. Only a few repurposed drugs have made it to clinical investigation. Metformin (anti-diabetic), somatostatin (statin), and mifepristone (anti-progestogen) increase the disease-free survival of patients suffering from ccRCC compared to the approved first-line treatment [20,21], shown by a retrospective analysis of a clinical evaluation in ccRCC. Nevertheless, none of these drugs repurposed as monotherapies have been investigated further or approved for the treatment of ccRCC, limiting the treatment options and potential success in patients. Metformin combined with vandetanib (VEGFR2) was discontinued because of a terminated drug supply by the pharmaceutical company (NCT02495103). Further studies monitoring kidney disease showed a tremendous need to adjust the metformin dose in a personalized fashion [7,22,23]. Inter- and intra-patient heterogeneity, together with the development of acquired drug resistance and single-drug dose-limiting toxicities, remain significant obstacles for the successful treatment and cure of RCC. Well-designed drug combinations may help overcome the current limitations of ccRCC therapy by challenging the robust biological processes [24,25]. Combination therapies can overcome complications linked to side effects and the induction of drug-related resistance due to non-overlapping mechanisms of actions [26,27]. Moreover, multitarget combination therapies can effectively inhibit critical oncogenic signaling pathways. The mutations and deregulations of signaling pathways link to the robustness and adaptability of complex biological systems that favor compensatory mechanisms, which can be exploited by tumors. Targeting those (compensatory) signaling pathways at multiple levels can enhance efficacy and therapeutic selectivity [26,28].

Recent clinical trials (e.g., CheckMate-214 [29], IMmotion151 [30]) have focused on combinations of tyrosine kinase inhibitors and immunotherapeutic regimens to treat ccRCC and overcome acquired drug resistance. However, the application depends on the efficacy of each drug as a monotherapy and a scheduling model [29,30], which is inferior regarding dose-limiting, drug half-life and off-target effects. Furthermore, there is high interest in combining repurposed drugs with anti-cancer drugs or other non-oncology drugs to broaden the spectrum of valuable treatment options. Hence, developing drug combinations impedes choosing the ‘right’ drugs to obtain a highly efficient synergistic effect with an alternate purpose [31,32].

Another critical obstacle in RCC management is nephrotoxicity-induced kidney failure due to cancer treatment. Since cisplatin, a platinum-based drug, was approved for the treatment of various cancer types, it has become the most prominent metal-based compound currently used in clinical settings. However, cisplatin causes severe side effects, with nephrotoxicity being the most common and intriguing problem.

Therefore, new metal-based drugs have been developed. One prominent example is Rapta-C, which represents an innovative anti-cancer therapeutic and is a better-tolerated alternative to cisplatin [33]. Rapta-C is a ruthenium(II)-based compound with amphiphilic properties [34]. Rapta-C exhibits anti-angiogenic, anti-cancer, and anti-metastatic activities through protein and histone-DNA alterations [33,35,36,37,38,39]. Compared with other ruthenium-based drugs, Rapta-C is strikingly competitive, especially when administered in combination with other drugs [35].

In this study, we selected seven repurposed non-oncology drugs and screened for multidrug combinations to potentiate the activity of Rapta-C. Using our validated phenotypic approach [25,40,41,42], we identified a low-dose, synergistic, optimized drug combination (ODC). This ODC selectively targeted cancer cells and was active in sunitinib-resistant cells, reducing the ATP production by 50%. The ODC contained Rapta-C and erlotinib (anti-cancer), metformin (anti-diabetic) and parthenolide (anti-inflammatory). We evaluated the cellular and molecular changes induced by the treatment with this drug combination, which demonstrated genetic and metabolic regulations. These results showed significant alterations in the cellular metabolism, notably lipid metabolism. Furthermore, our data revealed dysregulations in cell attachment and transcription. In vitro validation in complex heterotypic 3D co-cultures and murine organoid-like cultures led to an in-depth analysis of the anti-cancer efficacy regarding a favorable safety/toxicity profile at the cellular level, suggesting a good translatability of the ODC.

## 2. Materials and Methods

### 2.1. Cell Culture

Three distinct human ccRCC cell lines, A498, Caki-1 and 786-O and non-cancerous HEK-293T (human embryonic kidney) cells, were purchased from ATCC. The cancer cell lines were isolated from the epithelial part of cancer lesions, with A498 being isolated from a primary ccRCC lesion, Caki-1 from a skin metastasis, and 786-O from a primary clear cell adenocarcinoma. Subsets of A498, Caki-1, and 786-O RCC cells were split and chronically treated with 1 µM sunitinib to obtain sunitinib-naïve and sunitinib-resistant cells, referred to as -SR. Normal human adult dermal fibroblasts (NHDFα) were purchased from Vitaris (Baar, Zug, Switzerland). Prof. AW Griffioen (Angiogenesis Laboratory, Vrije Universiteit Amsterdam, 1081 HV Amsterdam, The Netherlands) generously donated human immortalized endothelial cells (ECRF24). Immortalized epithelial cells isolated from the renal cortex and proximal tubule (RPTEC/TERT1) as well as immortalized peripheral blood monocytes (THP-1) and T cells (BCL2 Jurkat) were purchased from ATCC.

Cells were cultured at 37 °C in either DMEM (A498, Caki-1) or RPMI medium (786-O and HEK-293T, THP-1, Jurkat) supplemented with 10% fetal bovine serum (S1810-500, Biowest, Nuaillé, France) and 1% penicillin/streptomycin (4-01F00-H, Bioconcept, Basel, Switzerland,). NHDFα cells were cultured in special fibroblast medium (C-23110-PRO, Vitaris) and ECRF24 in 50:50 DMEM and RPMI medium in flasks coated with 0.2% gelatin. RPTECs were cultured in epithelial cell medium (4101, ScienCell Research, Carlsbad, CA, USA).

Cells were tested for mycoplasma contamination frequently and have been authenticated by Microsynth AG (Balgach, St. Gallen, Switzerland). Cell line identity was confirmed with STR systems from Promega (Zurich, Switzerland) and subsequent database comparison.

### 2.2. Drugs

3-Hydroxytyramium (S15944309, Merck, Zug, Switzerland), diclofenac (D6899-10G, Sigma-Aldrich, Zug, Switzerland), and haloprogin (FD131, SynChem, Altenburg, Germany) were purchased, as indicated. Rapta-C was synthesized using a method presented elsewhere in the literature [43]. Acetylsalicyl acid (HY-107831), disulfiram (HY-B0240), famotidine (HY-B0377), ivermectin (HY-15310), metformin (HY-17471A), parthenolide (HY-N0141) and verapamil (HY-A0064) were purchased through MedChemExpress (Monmouth Junction, NJ, USA). Erlotinib-HCl was purchased from LC laboratories (Woburn, MA, USA). 3-Hydroxytyramium, diclofenac and metformin were dissolved in sterile ultrapure water. All other drugs were dissolved in sterile DMSO. Dependent on the solubility, drug stock solutions were prepared for long-term storage at −80 °C (Table S11). Sunitinib (Pfizer), was dissolved in sterile DMSO (Sigma-Aldrich, D8418-50 ML) and used as positive control at 0.01% in cell culture medium.

### 2.3. Heterotypic 3D Co-Cultures

Heterotypic 3D co-cultures of 700 ccRCC, 200 NHDFα and 100 ECRF24 cells [44] were established without the addition of extracellular matrix components or supplemented with 0.5 mg/mL collagen type I (A1048301, Sigma Aldrich). Those co-cultures were further supplemented with 35 Jurkat and 70 HP-1 cells [44] in experiments measuring the impact of the ODC_REMP_ treatment on those immune cell subsets. To promote spheroid formation, 80 µL/well were seeded in a round-bottomed 96-well plate with a cell-repellent surface (Cellstar^®^ 650970), or in 96-well round bottomed plates (353077, Beckton Dickinson, Nyon, Switzerland) coated with poly-2-hydroxyethyl methacrylate (P3932-10G, Sigma Aldrich) and centrifuged at 4 °C for 2 min at 1200 rpm. Drug treatments on co-cultures were initiated 48 h after spheroid formation.

### 2.4. Cell Viability Assay

Drug treatment activity in 2D and 3D cell culture models was measured using the CellTiter-Glo^®^ cell metabolic activity (ATP) assays (G7572 and G9683, Promega, Madison, WI, USA), according to the manufacturer’s instructions. Bioluminescence readout was performed using a BioTek Cytation 3 and Gen5 Image software version 3.04 at default settings.

### 2.5. RNA Sequencing

An RNA easy^®^ Plus Kit (74134, Qiagen, Hilden, Germany) was used to isolate RNA following the manufacturer’s instructions. The RNA quality control was performed as well as library preparation through TruSeqHT Stranded mRNA (Illumina). An Illumina HiSeq 4000 System using the 100 bp single-end reads protocol was applied for the sequencing experiments. A quality control was added with FastQC v.0.11.5. The reads were aligned to the human genome (UCSC hg38) using STAR v.2.5.3a software [45]. With PicardTools v.2.9.0, the biological quality control was incorporated and the HTSeq v.0.9.1 was utilized for raw count acquisition [45]. Normalization and differential expression analysis were performed using the R/Bioconductor package edgeR v.3.24.3 [46]. A general linear model, negative binomial distribution, and quasi-likelihood F test were applied to assed the statistical significance. Genes with a fold change >2 and *p*-value < 0.05 (with a false discovery rate of 5%) were considered differentially expressed.

Genes up- and downregulated after ODC treatment were analyzed according to the gene ontology enrichment analysis in Enrichr [47]. The RNA-Seq data have been deposited in the NCBI Gene Expression Omnibus [48] with GEO Series accession GSE174740.

### 2.6. Metacore Analysis

MetaCore^TM^, a web-based analytical tool of Cortellis^TM^ and hosted by Clarivate Analytics [49], was used to perform network and pathway analysis of RNA sequencing data to obtain a graphical output. This analysis tool enabled the development of a molecular understanding of the genetic alterations after drug combination treatment in Caki-1-SR cells in comparison to sham-treated Caki-1-SR cells. The analysis was performed focusing on 10–30 nodes in one pathway or network, to comprehensively mine for the underlying pathway alterations.

### 2.7. Analysis of the Lipid Classes

Sample preparation and LC-HRMS/MS analysis was performed following the same procedure as previously described by Rausch et al. [50]. The MS data were converted from RAW (Thermo) standard data format to .mzXML format using the MSConvert software, part of the ProteoWizard package [51]. The converted files were treated using the MZMine software suite v. 2.38 [52]. A more detailed explanation on data treatment is given in the Supplementary Information S1. The data are deposited on GNPS and can be accessed through doi:10.25345/C5P53K.

### 2.8. Flow Cytometry Analysis

The 3Dcc^imm^ were characterized through flow cytometry analysis. The 3Dcc^imm^ spheroids were dissociated using AccuMax-solution to obtain single-cell suspension and antibody-labelling was performed on ice for max. 1 h. Fluorophore-coupled antibodies are listed in Table S12 and were purchased from BD Bioscience (San Jose, CA, USA) or Biolegend (London, United Kingdom). Annexin V-APC (640920) and propidium iodide solution (421301) were used to analyze apoptosis and cell cycle distribution. Both chemicals were purchased from Biolegend and used following the instructions given by the manufacturer. The cells were analyzed on a Beckton Dickinson (BD) LSRFortessa (5 lasers; Franklin Lakes, NJ, USA) and BD FACSDiva^TM^ v9 software (San Diego, CA, USA).

### 2.9. Organoid Generation

Experiments including Swiss nu/nu mice (NU(Ico)-Foxn1nu) were performed in accordance with the Institutional Ethical Committee of Animal Care in Geneva and the Swiss Cantonal Veterinary Office (authorization number GE-100-20). Both female and male mice were obtained at an age of 6–8 weeks from Charles River (Écully, France). A total of 5 × 10^6^ Caki-1 or Caki-1-SR cells were harvested and re-suspended in 100 µL of DMEM medium supplemented with 1% FCS. Cells were inoculated subcutaneously in the left flank. Tumor growth was monitored for 21 days. After sacrificing the animals, tumors were resected and dissociated enzymatically and mechanically to obtain tissue chunks of <1 mm^2^. Cell-tissue chunks were maintained for 6 days to promote cell aggregation, proliferation, and the natural formation of spheroids and organoid-like constructs in StemPro medium [53]. To perform the analysis of treatment response, these aggregates were re-dissociated to obtain a single-cell suspension and seeded, following the same protocol as for 3D cultures from cell lines distributing 1000 cell per well.

## 3. Results

### 3.1. Phenotypic Synergy Screen Identifies Drug Hits for Combinatory Treatment in ccRCC Cell Lines

We intended to block the cellular responses and signal transduction of ccRCC at varying levels in our approach. Therefore, we included drugs in the initial set that bound to extra-, intra-cellular and nuclear, i.e., DNA targets (Table 1). Chemotherapeutics bind and alter DNA, and at present, ruthenium-based drugs have proven to be strong candidates [54,55]. Rapta-C, an experimental ruthenium (II)-based compound, binds to the histone proteins in chromatin, exploiting favorable anti-cancer activity, further demonstrated to be favorable for drug combination studies [35].

Intending to identify drugs that might interact synergistically in combination with Rapta-C, we selected eleven repurposed drugs, based on the following criteria: (i) reported anti-cancer activity; (ii) validation via the ReDo project [14]; (iii) literature information [4,6,7,13,15,69,70]; and (iv) known combinations with cisplatin or with ruthenium(II)-based compounds (Table S1).

In the first step, we evaluated the dose-related cytotoxicity of each drug to initiate the synergy screen by creating drug response curves (Figures S1–S3). This was performed in parallel in human treatment-naïve and chronically treated ccRCC cells with sunitinib (-SR), as well as in non-cancerous HEK-293T and RPTECs of kidney origin.

The dose–response curves were used to determine the doses needed to initiate the screen. Two doses were chosen to calculate the dose-dependent effects of each drug (Table S2). The concentrations tested aligned with the reported maximal plasma dose (MPD) of (i) mice during preclinical testing or (ii) patients during clinical testing or after approval. The concentrations were related to the standard application for the initial indication. We performed the experiments in ccRCC cell lines and measured the cellular metabolism (ATP levels) compared to the sham-treated control (CTRL, 0.01% DMSO in culture medium). The drug and dose-dependent responses were similar for all ccRCC cell lines.

**Figure 1:**
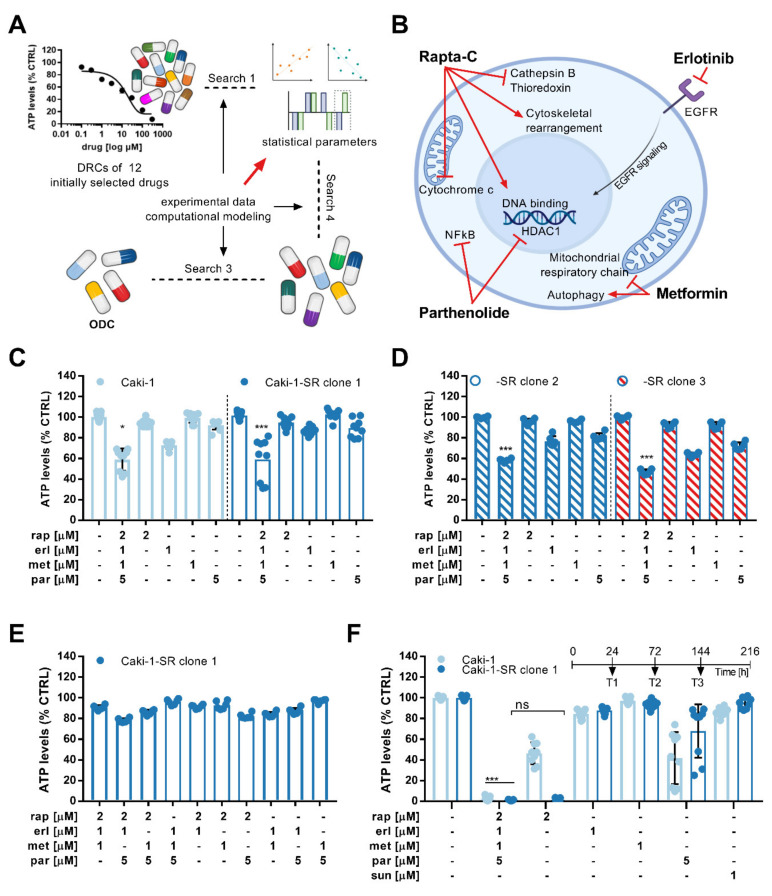
Optimization of a multidrug combination and its activity in 2D cell cultures. (**A**) Schematic representation of the Therapeutically Guided Multidrug Optimization method to screen for multidrug combinations. An initial set of 12 drugs was selected and drug-response curves (DRCs) were generated to visualize the single-drug effects. In three searches, experimental and computational modeling were used to generate data facilitating the calculation of statistical parameters which are needed to guide drug inclusion and exclusion between the searches. Following this iterative process, a four-drug combination was designed and optimized (ODC). (**B**) This ODC contains Rapta-C, erlotinib, metformin and parthenolide (ODC_REMP_). The known targets for ODC_REMP_ drugs are presented in the schematic of a cancer cell. (**C**) Bar graphs demonstrating the ATP levels (viability measure) of Caki-1 and Caki-1-SR clone 1 cells in response to the drug combination and monotherapy treatment. The error is presented as the standard deviation. Significance was calculated for *N* = 3 independent experiments using a one-way ANOVA test; * *p* < 0.05, *** *p* < 0.001. (**D**) Measure of the ATP levels after ODC_REMP_ and monotherapy treatment in two Caki-1-SR clones 2 and 3 established following different protocols to induce resistance to sunitinib. Clone 2 was established, maintaining the chronic treatment of 1 µM sunitinib for more than 30 weeks. Clone 3 demonstrated intrinsic resistance after one single treatment of 10 µM sunitinib. The error is presented as the standard deviation. Significance was calculated for *N* = 3 independent experiments using a one-way ANOVA test; *** *p* < 0.001. (**E**) Upon the removal of one or two drugs at the time from the combination, the anti-cancer efficacy decreased significantly. (**F**) Retreating Caki-1 and Caki-1-SR cells 3 times, each 72 h, (time line above the graph) reduced the ATP levels significantly to values <5% compared with the untreated control. The error is presented as the standard deviation. Significance was calculated for *N* = 3 independent experiments using a one-way ANOVA test; *** *p* < 0.001.

A phenotypic screen called Therapeutically Guided Multidrug Optimization [40,41,42,71] (TGMO, Figure 1A) was used to identify drug–drug interactions between the twelve drugs administrated at two doses. Drug–drug interactions were compared to interactions reported in the literature (Table S3).

Following the TGMO design, a small number of experimental data points were gathered in searches 1–3 and fed into a model mathematically describing single drug effects, dose-related effects, two-drug interactions, and the relationship between the drug combination input and output activity of each possible two-drug combination (Figures S4 and S5) [25]. Regression coefficients are the final output of the linear regression calculation, which can be used to ascertain synergy, additivity or antagonism between drugs.

In the course of the TGMO, multiple combinations varying in the number of drugs were tested, ranging from twelve to two drugs. Search 1 was dedicated to determining the drugs not adding efficacy to the combinations or presenting an antagonism to most other candidates. Transient from search 1 to search 2, five drugs were removed, and the remaining were screened again. In search 2, we included the therapeutic window, which describes the difference of the combined cytotoxic effect in non-cancerous cells (RPTECs) and ccRCC cells, eliminating a further three drugs. In search 3, four drug candidates per cell line were tested to determine the final drug combination.

The three consecutive search rounds led to identifying an optimized cell-type-specific drug combination (Figures S4 and S5) in Caki-1 sunitinib-naïve (SN) and sunitinib-resistant (-SR) cells [50]. This combination consisted of Rapta-C, erlotinib, metformin and parthenolide, and was further abbreviated as ODC_REMP_ (Figure 1B). Mathematical and statistical information accompanying the TGMO search showed underlying synergy (CI = 0.65 [72]; Figure S6) between the drugs and a robust model accuracy represented through a coefficient of multiple determination (Figure S7). The combination presented a similar efficacy in reducing the ATP levels of both SN and SR cells (>40%), and in Caki-1-SR cells, the combined activity was more potent than the single-drug activities (Figure 1C).

### 3.2. Activity Validation of the ODC

We discovered that sunitinib-naïve and -resistant ccRCC cells of primary origin (A498, 786-O) were predominantly insensitive to ODC_REMP_ (Figure S8). This result suggests that the anti-cancer activity in Caki-1(-SR) cells is based on triggering molecular targets particularly present in these cell lines. Treating non-cancerous cells originating from non-malignant tissue, i.e., human kidney tubular epithelium (RPTEC), endothelium (ECRF24), and fibroblast (NHDFα; Figure S9), revealed that the ATP levels remained stable upon treatment. In addition, we included immortalized immune cell lines, i.e., BCL2-Jurkat CD4+ T cells and THP-1 monocytes. The ATP levels of Jurkat and THP-1 cells cultured separately in 2D cultures decreased by approximately 50%.

Exposing Caki-1-SR clones 2 and 3, which have different genetic backgrounds, to ODC_REMP_, the activity of the combination was maintained (Figure 1D). At the same time, the removal of one or two of the drugs from ODC_REMP_ reduced the activity. Dose increases in ODC_REMP_ increased the activity in RPTECs (Figure 1E and Figure S10).

The results described above were obtained after a single 72 h incubation of cells with drugs. Another two consecutive times (72 h), retreatment of the same cells led to a further significant decrease in the ATP level (Figure 1F).

### 3.3. RNA Sequencing Reveals the Upregulation of Genes Related to Apoptosis, Cell Adherence and Metabolism upon ODC_REMP_ Treatment

**Figure 2:**
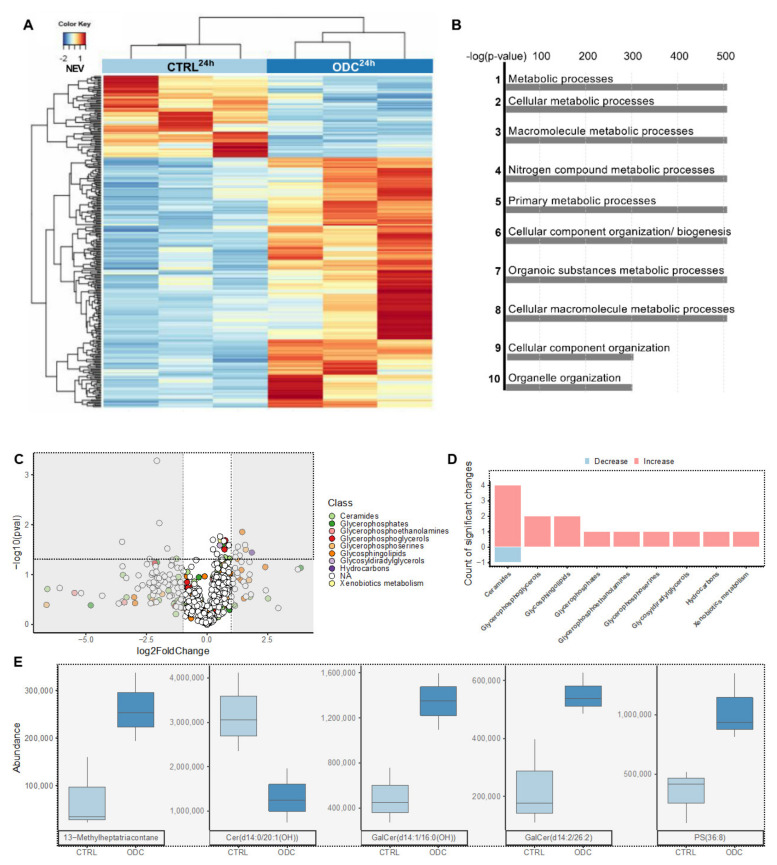
RNA sequencing and LC-HRMS/MS metabolomics analysis of Caki-1 cells treated or not with ODC_REMP_. (**A**) Heat map representing the significant differential expression of 93 genes, comparing sham-treated Caki-1-SR (CTRL) and ODC_REMP_-treated Caki-1-SR cells (REMP). The treatments were applied for 24 h before the analysis. The normalized expression value (NEV) is represented by a color scale, with blue for a low expression and red for a higher expression value. (**B**) Pathway enrichment analysis showing that the gene ontology terms correspond to significant alterations in metabolic processes. The top 10 significantly dysregulated pathways are listed. (**C**) Volcano plot of the 624 features (m/z at retention time) obtained after metabolomics treatment of the LC-MS data in positive mode (Figure S14 and Table S9). Dots are colored according to the annotated chemical class. Dots included in the light grey rectangles represent an absolute log (fold change) higher than 1. Dots included in the black dashed rectangle represent significant changes (*p* < 0.05) and are detailed in (**D**), classes with significant changes. The class with highest number of significant changes (four increased and one decreased) was ceramide, with other significantly increased phospholipids classes. (**E**) Normalized peak areas of the five annotated features both significantly increased or decreased with an absolute log (fold change) higher than 1 (present in black dotted and grey rectangles). Features were labelled according to RefMet [76]. An overview of all ceramides with either *p* < 0.05 or abs (log(fold change)) > 1 is given in Table S9.

To study the consequences of ODC_REMP_ treatment on RNA expression (Figure 2A), we incubated Caki1-SR clone 1 cells for 24 h with ODC_REMP_ to capture the onset of gene down- and upregulation. Significant differential expression was detected for 284 genes (raw *p*-value < 0.05 and FC ≥ 2) between the control conditions (untreated Caki-1-SR cells; Caki-1-SRCTRL) and Caki-1-SR cells treated with ODC_REMP_. These alterations included a cluster of 22 genes (*p*-value with false discovery rate (FDR) < 0.05 and FC ≥ 2) with 2 being down- and 20 upregulated corresponding to cell signaling and transcription processes (Table S4). The functions of these genes were attributed to chemotaxis, metabolic pathways and DNA damage repair (Figure 2B, Figures S11 and S12). Following network analysis in MetaCoreTM, variations in the regulation of apoptosis, cell adherence, cytoskeletal rearrangement, and transcription were detected (Figure 2C). The most significant changes, highlighted through the MetaCoreTM network analysis, were found in the necrosis factor-kappa B (NF-κB) signaling pathway between NF-κB and the upstream located inhibitory kinase of NF-κB located upstream (44.4%; 5.166–22), together with a positive regulation of NF-κB transcription factor activity (48.1%; 8.049–20; Tables S5 and S6).

The targets, detected through RNA sequencing, were not necessarily part of the signaling pathways with which these drugs are known to interfere. Therefore, we used an in silico prediction tool (SwissTargetPrediction [73]) to determine whether these targets might be off-targets with suitable binding sites. We collected the canonical SMILES codes (Table S7) for the drugs erl, met and par from PubChem within the ODC_REMP_ and inserted them in the search tool. Rapta-C has a complex structure; therefore, the generation of the SMILES code was difficult and had to be adapted from previous studies [33,34,74,75] to fit the prediction tools. We selected all targets with a binding probability > 0.05 and ordered them alphabetically (Table S8). This step revealed that RNA sequencing showed the interplay of the drugs and the regulation of non-obvious targets. The output of the prediction tool referenced four targets for Rapta-C with a binding probability of 0.1016.

In parallel, we performed Western blot experiments to determine the protein expression of hypoxia-inducible factor-1α, HDAC1/2, Rho-associated protein kinase, as well as CD41 and CD44. Both CD41 and CD44 are proteins related to epithelial–mesenchymal transition, which occurs when cells of endothelial origin (as the Caki-1-SR cells) lose cell–cell adhesion. The analysis was performed after 24 h treatment; however, no significant changes were detected (Figure S13; Tables S6 and S8).

### 3.4. ODC_REMP_ Treatment Has an Impact on the Metabolism of Ceramide and Glycerophospholipids in ccRCC Cells

The effect of the ODC_REMP_ treatment on the cellular metabolism detected through RNA sequencing was investigated further through the analysis of the cell and drug metabolism by high-resolution tandem mass spectrometry (LC-HRMS/MS). We focused on drug metabolites and apolar cell metabolites because of the reported effects of metformin and parthenolide on the lipid metabolism [77,78,79].

After 24 h of incubation with ODC_REMP_, the culture medium containing the drugs (supernatant) was separated from the cells. The cells were quenched, and metabolites were extracted using methanol (cell extract; Figure S14A). In the supernatant (Figure S14C, right graph), all peaks corresponding to the four drugs of the ODC_REMP_ were detected (compared with standard solutions; Figure S14B). For sunitinib, two peaks were detected in the supernatant and the extract, corresponding to the two stereoisomers of sunitinib [50] (Figure S14C,D). However, in the cell extract, only erlotinib was detected together with sunitinib isomers [80]. The reversible conversion from (*Z*)-sunitinib to (*E*)-sunitinib [34,80] occurs through light exposure (Figure S14C). We have previously reported on both (*Z*)- and (*E*)-sunitinib isomers in the cell interior after sunitinib-resistance induction [50].

We also observed alterations of metabolites not related to the drugs themselves following the ODC_REMP_ treatment (Figure 2B). Focusing on the lipid classes, we detected significant increases in ceramides and lipids with glycerophosphate residues (Figure 2C). Inspecting these alterations per lipid class showed a rise in sphingolipid derivatives after ODC_REMP_ treatment (Figure 2D). Within the most significantly changed sphingolipids were the main classes of ceramides, glycosphingolipids and glycerophosposerines (Figure 2E and Table S9).

**Figure 3:**
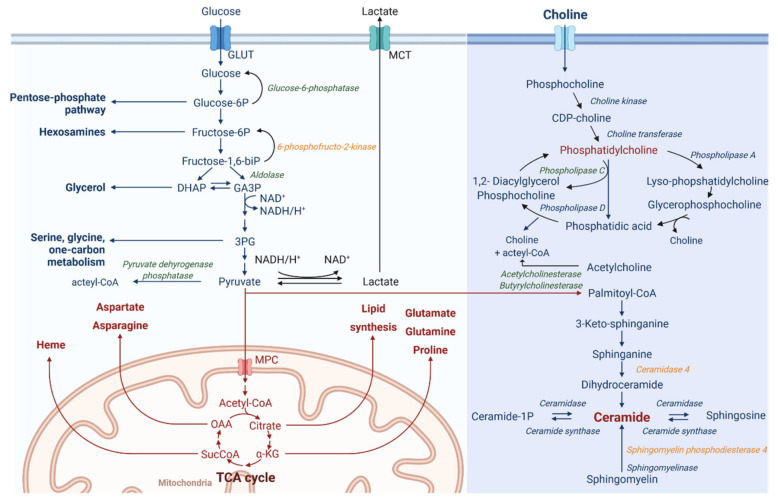
Schematic representation of metabolic pathways targeted by ODC_REMP_. RNA sequencing and LC-HRMS/MS data revealed a strong impact on metabolic pathways after ODC_REMP_ treatment. These pathways include glycolysis, choline and ceramide metabolism. Enzymes detected to be upregulated after ODC_REMP_ treatment (in italics and orange), downregulated enzymes (in italic and green), and upregulated metabolites (written in red). Pyruvate can be used for the enzymatic conversion of palmitoyl-CoA, which is the primary molecule to initiate the de novo synthesis of ceramide. Molecules of the ceramide class were significantly upregulated, as well as the two enzymes ceramidase 4 and sphingomyelin phosphodiesterase 4. Pyruvate is also the substrate for pyruvate dehydrogenase phosphatase producing acetyl-CoA. This figure was created with BioRender.com. Legend: αKG, α-ketoglutarate; 1/6(bi)*p*, 1/6 (bis)phosphate; 3PG, 3-phosphoglyceric acid; CDP-choline, cytidine 5′-diphosphocholine; CoA, coenzyme A; DHAP, dihydroxyacetone phosphate; GA3P, glyceraldehyde 3-phosphate; GLUT, glucose transporter; MCT, monocarboxylate transporter; MPC, mitochondrial pyruvate carrier 1; NAD, nicotinamide dinucleotide; OAA, oxaloacetate; SucCoA, succinyl-CoA; TCA, citric acid cycle.

Connecting between gene expression and lipophilic metabolite patterns (Figure 3) revealed that ODC_REMP_ influences ceramide, choline and glucose metabolism. This connection has been performed based on the single read-outs linking the metabolites with the corresponding enzymes. Glycolysis in cancer cells is often used to produce lactate (Warburg effect [81,82]) from glucose. Our data suggested that the expression of glucose-6-phosphatase is downregulated; this phosphatase reconverts glucose-6-phosphate to glucose. Downstream, pyruvate can be enzymatically transformed to acetyl-CoA or lactate. During aerobic glycolysis, acetyl-CoA is produced. However, our RNA sequencing data revealed a downregulation of the pyruvate dehydrogenase phosphatase enzyme, which converts pyruvate to acetyl-CoA. This indicates that after ODC_REMP_ treatment, cells, in turn, might induce the production of lactate and lipid synthesis. Such behavior can also be seen with the Warburg effect, which describes the modified cellular metabolism in cancer cells favoring lactate production from glucose in an aerobic environment.

Mass spectrometry analysis detected a substantial increase in phospholipid metabolism. By linking this result with RNA sequencing data, we detected a significant downregulation (FC ≥ 2, *p*-value with FDR < 0.05) of phospholipase C, the enzyme converting phosphatidylcholine to 1,2-diacylglycerol. There are two ways of supplying choline for this reaction, i.e., extracellular uptake and production during phosphatidylcholine metabolism. Further downstream in the metabolism of phosphatidylcholine, the enzymes acetylcholinesterase and butyrylcholinesterase were downregulated. These enzymes use acetylcholine to produce choline and acetyl-CoA.

Upon ODC_REMP_ treatment, the presence of ceramides was significantly increased (Figure S15 and Table S9). This appears to be forced by the significant upregulation of the enzymes ceramidase 4 and sphingomyelin phosphodiesterase 4, leading to ceramide synthesis from palmitoyl-CoA sphingomyelin.

To associate the cellular changes after ODC_REMP_ treatment with physicochemical properties and pharmacokinetics, we used SwissADME [83]. SwissADME is an in silico prediction tool developed by the Swiss Institute of Bioinformatics [83]. The prediction did not offer information on absorption, distribution, metabolism, and excretion after the combined application of the drugs. Therefore, we could not obtain an accurate model, but characterized each drug separately (Table S10). The predictions for passive human gastrointestinal absorption and blood–brain barrier permeation both consisted in the readout of the BOILED-Egg model [84] (Figure S16). All compounds in the ODC_REMP_ are absorbed in the intestine presented through the outer area. Three compounds may pass the blood–brain barrier (yellow area) accessing the brain (red circles). In general, the majority of drugs in the ODC_REMP_ are poorly absorbed (red dots).

### 3.5. ODC_REMP_ Activity in Complex Heterotypic 3D Co-Cultures

In order to mimic the tumor microenvironment, we have previously developed complex heterotypic 3D co-cultures (3Dcc) closely representing the biological characteristics of ccRCC [44]. We prepared scaffold-free 3Dcc containing Caki-1-SR, NHDFα and ECRF24 cells and, in parallel, the same co-cultures supplemented with 5% Jurkat and 10% THP-1 cells (3Dcc^imm^).

**Figure 4:**
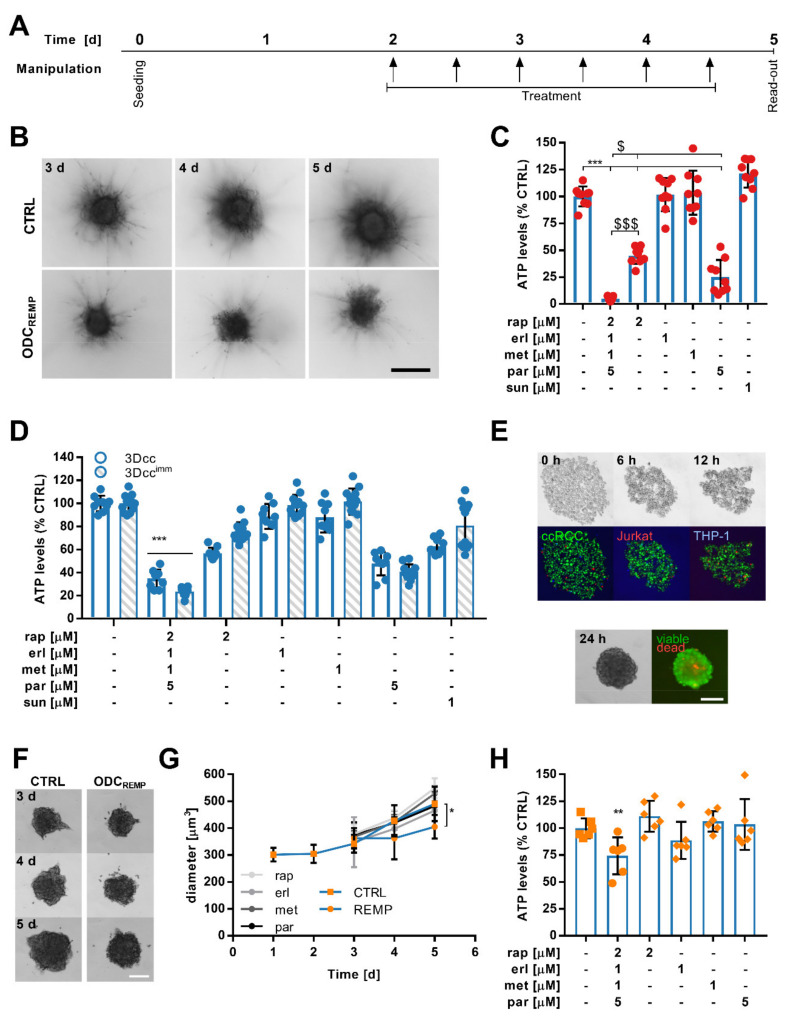
Activity of ODC_REMP_ in in vitro and ex vivo organoid-like heterotypic 3D co-culture systems. (**A**) Timeline of the treatment schedule used for translation in heterotypic 3D co-cultures from cell lines and resected Caki-1-SR clone 1 tumors. (**B**) Caki-1-SR clone 1-based 3D co-cultures (3Dcc) were established in a collagen-rich (0.5 mg/mL collagen type 1) environment to induce a motile phenotype and sprout formation. After three days (3 d) sprouts were formed, which were monitored until 5 d. Representative bright-field images show the difference between untreated (CTRL) and ODC_REMP_-treated spheroids. The scale bar corresponds to 300 µm. (**C**) Bar graphs demonstrating the energy loss (reduction in ATP levels) of Caki-1-SR clone 1 3Dcc spheroids cultured in a collagen-rich environment in response to ODC_REMP_ and monotherapy treatment. The error is presented as the standard deviation. Significance was calculated for *N* = 3 independent experiments using a one-way ANOVA test; * represents the comparison between all conditions, * *p* < 0.05, ** *p* < 0.01, *** *p* < 0.001. $ represents the comparison of the ODC_REMP_ to single monotherapies only, ^$^ *p* < 0.05, ^$$$^ *p* < 0.001. (**D**) Comparison of the treatment response of Caki-1-SR clone 1-based 3Dcc and co-cultures including immune cell lines (3Dcc^imm^). To obtain 3Dcc^imm^ spheroids, 5% Jurkat and 10% THP-1 cells were supplemented. The error is presented as the standard deviation. Significance was calculated for *N* = 3 independent experiments using a two-way ANOVA test; *** *p* < 0.001. (**E**) Representative bright-field and fluorescence images of the co-culture formation in times ranging from the moment of seeding (0 h) to 12 h. The cells were stained with CellTrackerTM dyes to monitor the movement during spheroid formation (green, ccRCC; red, Jurkat; and blue, THP-1). After 24 h, unstained spheroids were transferred from the culture well into a staining well including a solution of ethidium homodimer and calcein to visualize viable and dead areas in the spheroid (green, viable; red, dead). The scale bar corresponds to 200 µm. (**F**) Representative bright-field images of ex vivo organoid-like cultures derived from murine tumor tissue on day 3–5 (3–5 d) after seeding. Organoid-like cultures remained untreated (CTRL) or were exposed to ODC_REMP_ treatment following the schedule depicted in A. The scale bar corresponds to 200 µm. (**G**) Measurement of the diameter of ex vivo organoid-like cultured derived from murine tumor tissue. Significance was calculated for *n* = 6 independent organoid-like cultures using a one-way ANOVA test; * *p* < 0.05. (**H**) ATP levels of ex vivo organoid-like culture responses after repeated ODC_REMP_ and monotherapy treatment. The error is presented as the standard deviation. Significance was calculated for *n* = 6 independent organoid-like cultures using a one-way ANOVA test; ** *p* < 0.01.

We determined the effect of the ODC_REMP_ on (i) the production of the extracellular matrix and spheroid growth, (ii) the motility of cells, and (iii) the immune cell subsets. Considering the complex spheroid environment and the short half-life of the drugs (Table S1), we administered ODC_REMP_ six times, every 12 h, Figure 4A. Culturing the 3Dcc in a medium supplemented with 0.5 mg/mL collagen type 1 facilitated the formation of sprouts used for cellular movement (Figure 4B,C). Through adherence to the collagen network, cancer cells induced migration away from the core spheroid, which seemed to be accompanied by the motion NHDFα and ECRF24 cells. Even after the appearance of the motile phenotype, the anti-cancer efficacy of the ODC_REMP_ significantly reduced the ATP levels. Measuring the spheroid size (Figure S17A) revealed a significant decrease of 181.5 μm^3^ between the untreated and ODC_REMP_ spheroids. The diameters of the stable core spheroid (inner) and the motile margin (outer; Figure S17B) were shortened by >165 μm (inner: 169.5 μm; outer: 176.1 μm). The ODC_REMP_ significantly reduced the number of sprouts (Figure S17C), but not the length of the sprouts (Figure S17D).

The administration of ODC_REMP_ every 12 h potentiated ODC_REMP_ efficacy compared to the single treatment observed in 2D cell culture, actively reducing the production of ATP in both 3Dcc and 3Dcc^imm^ (Figure 4D). Similar results were observed in 3Dcc and 3Dcc^imm^ based on Caki-1 cells (Figure S18A). ODC_REMP_ treatment in 3Dcc and 3Dcc^imm^ based on Caki-1-SR clone 2 or 3 [50] revealed the same persistent activity (Figure S18B). Remarkably, the efficacy of parthenolide increased 4.4-fold in the Caki1-SR clone 2 and 3 cells.

In the next step, we established ex vivo organoid-like cultures from Caki-1-SR clone 1 tumors grown subcutaneously in Swiss nude mice (Figure 4F). Following the same ODC_REMP_ administration scheme (Figure 4A), its activity was represented by significantly diminished murine organoid-like culture diameter (Figure 4G) and ATP levels (Figure 4H).

### 3.6. Flow Cytometry Measurements Reveal Selective ODC_REMP_ Targeting in Cancer Cells

**Figure 5:**
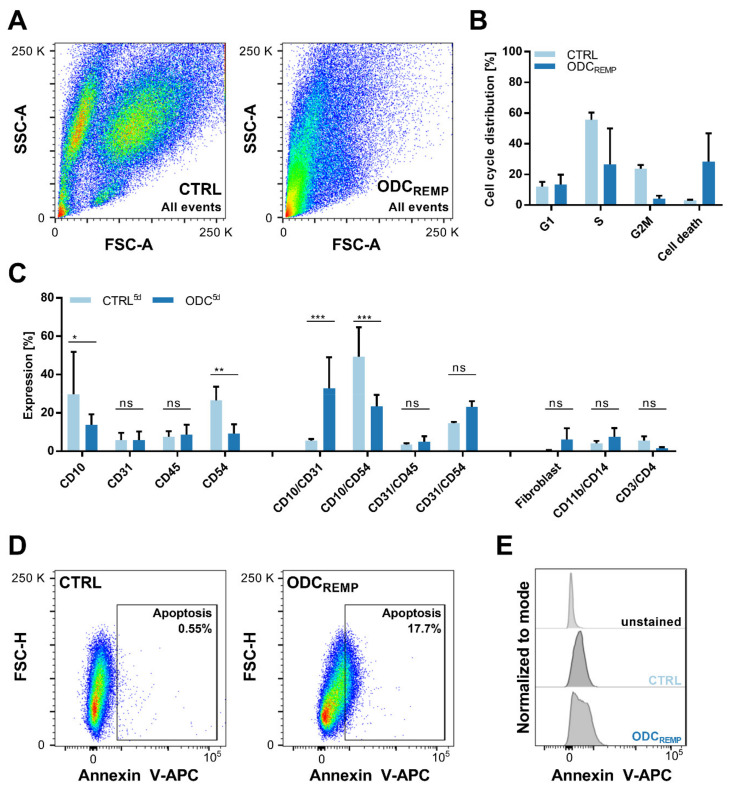
Flow cytometry analysis to define the selectivity of the ODC_REMP_ and its activity to arrest cell cycle progression. (**A**) Pseudocolor blots demonstrating the difference in the size and granularity (forward- and side-scatter signal; FSC and SSC, respectively) of 300 untreated (CTRL) and ODC_REMP_-treated 3Dcc^imm^ spheroids after dissociation into single-cell suspensions. (**B**) Propidium iodide staining was performed to detect the distribution of cells in the different stages of the mitotic cell cycle. (**C**) Expression of cell surface proteins shown through FACS analysis. The expression profile was captured after four and six repeated treatment administrations on day 5 (5 d) in comparison to the CTRL. Single and double protein expressions have been analyzed in comparison to cell surface proteins exclusively expressed on immune cells. The error is presented as the standard deviation. Significance was calculated for *N* = 3 independent experiments using a two-way ANOVA test; * *p* < 0.05, ** *p* < 0.01, *** *p* < 0.001. (**D**) Measurement of apoptosis through Annexin V staining demonstrating the increase in apoptotic cells after ODC_REMP_ treatment. (**E**) Comparison of the unstained control versus the untreated and ODC_REMP_-treated 3Dcc^imm^ spheroids shown in histograms.

The selectivity of the ODC_REMP_ treatment was analyzed through cross-validation in non-cancerous cell lines and flow cytometry analysis (FACS). The 3Dcc^imm^ were dissociated and monitored in single-cell populations, especially the immune cell cohort, quantifying the expression of characteristic cell surface proteins (Figure 5). Separation of the cells by size and the granularity showed that upon ODC_REMP_ treatment, the three distinct populations disappeared (Figure 5A). The recorded events shifted to the lower-left corner of the pseudocolor blot, indicating an increase in size, usually related to cell death. Therefore, we analyzed the cell cycle distribution, which confirmed an increase in cell death by 25.4% compared to the CTRL (Figure 5B). Simultaneously, we monitored the cell cycle upon ODC_REMP_ treatment. Fewer cells were detected in the S phase and the G2/M phase. The expression of cell surface proteins after six consecutive treatments with the ODC_REMP_ revealed that immune cell-related markers (CD45, CD3, CD4, CD11b, CD14) remained stably expressed, indicating that Jurkat and THP-1 cells were not affected by the treatment (Figure 5C). The co-expression of CD10 and CD31 was significantly increased, whereas the co-expression of CD10 and CD54 decreased. This suggested that endothelial cells and fibroblasts resist the treatment, whereas cancer cells are targeted selectively. Interestingly, a slight upregulation of the double expression of CD31 and CD54, an indicator of cell anergy [85], was detected. The evaluation of Annexin V, a specific marker for apoptosis, showed that 17.7% of the cells collected after 6x treatment underwent apoptosis (Figure 5D,E), which was correlated with the result of the cell cycle distribution.

## 4. Discussion

Multiple hurdles in drug discovery have led to the rapid development of alternative strategies, such as drug repurposing [3,6,7,14,70,86,87]. In this study, we optimized a synergistic four-drug combination composed of repositioned (other cancer types) and repurposed (diabetes, inflammation) drugs for the treatment of ccRCC (Figure 1). Drug doses used in the combination were 1.6- to 2.3-fold smaller than for their initial indication.

Rapta-C is an investigational drug; therefore, plasma doses are not available. However, compared to other in vitro studies, the dose was over fivefold lower [35]. The optimized reduction in the applied doses suggests that systemic administration in translation (in humans) may be achieved.

With the use of our phenotypic TGMO method, the first hit in the screening was metformin, previously reported as a prominent candidate for cancer therapy [12,23,88,89], usually applied as monotherapy at high doses [90]. It has been shown that metformin can further be used as a sensitizing agent to enhance the efficacy of chemotherapy in colorectal cancer cells [91]. The doses used to obtain this effect were 6–8-fold higher than those applied in our study. Similarly, calculating the maximal concentration of metformin in blood plasma indicated a 1.6-fold reduction in the dose in our experimental settings. Analyzing the drug–drug interactions revealed a strong synergy between erlotinib and metformin, which aided in diminishing the concentration.

We identified synergistic drug–drug interactions between erlotinib and parthenolide. Parthenolide is a natural product isolated from the plant feverfew (Tanacetum parthenium) with known anti-inflammatory and anti-cancer properties [65,66,92] targeting NF-κB and histone deacetylases (HDAC). Our previous study reported a drug combination of two HDAC and two tyrosine kinase inhibitors [41], highlighting the critical role of HDAC blockade in RCC treatment. Rapta-C and parthenolide both bind to HDACs, which confirms the dependency of RCC progression on the functionality of these enzymes, hence making them desirable targets. In general, Rapta-C is superior to other chemotherapeutics [33,37,38] and a favorable candidate for drug combinations [35,37,38,93]. Synergistic interactions of Rapta-C and erlotinib have been described in human endothelial cells and human ovarian carcinoma, demonstrating vigorous anti-cancer and anti-angiogenic activity [35].

Through RNA sequencing, we detected alterations in gene expression levels, which indicated a significant upregulation of apoptosis and cytoskeletal arrangement (Figure 2). Dysregulation of cell adherence [40] seemed to be impaired through Rapta-C [35,94] and parthenolide [92,95]. Treatment with parthenolide corresponded to the reduced formation of metastasis after the reduced expression of vimentin [95]. The presence of detyrosinated tubulin, a post-translational modification of α-tubulin, decreased cancer cell reattachment. We detected an upregulation of apoptosis-related proteins, i.e., Bcl-2, Bax, and caspases, which are generally downregulated in Caki-1 cells chronically treated and resistant to sunitinib [50]. Another protein involved in apoptosis is ROCK1, and although our results were non-significant, a potential upregulation can be seen (Figure S13). These results further reveal a downregulation after ODC_REMP_ treatment of HDACs and HIF-1α, which are targets of Rapta-C and parthenolide. Proteins potentially related to endothelial–mesenchymal transition, i.e., TGF-β signaling, and β-catenin mediated signaling, are upregulated (Figure S11). However, further investigation is required to confirm this observation. ODC_REMP_ treatment induced apoptosis in the cancer cells. Overcoming resistance to sunitinib is further linked to the upregulation of autophagy, which was downregulated in Caki-1-SR cells [50]. Genes linked with inflammatory responses were downregulated after 24 h of ODC_REMP_ treatment, suggesting that sunitinib-induced resistance is overruled, enhancing signaling through NF-κB. Makhov et al. documented that resistance to sunitinib is mediated through ER stress responses in ccRCC cells. Inducing a cascade of signaling molecules, i.e., pro-tumorigenic cytokines interleukin-6 (IL-6), IL-8 and tumor necrosis factor-α (TNF-α), induces the NF-κB transcriptional survival program, protecting tumor cells against cell death. These dependencies have been demonstrated both in vitro and in vivo [96], hence revealing that the downregulation of these factors impairs sunitinib resistance.

Our results imply a strong correlation between ODC_REMP_ treatment and the regulation of metabolic pathways. After 24 h of treatment, a significant increase in sphingolipids was measured (Figure 2 and Figure S15). The evidence is clear that ODC_REMP_ has an impact on glycolysis and lipid synthesis (Figure 2 and Figure 3). A glucose metabolism-reducing activity has been described for metformin [78], which is associated with lipid metabolism.

To fully elucidate the mechanism of action of the ODC_REMP_, further experiments are needed, but because four medications act simultaneously, it might very well be that the allocation of a single drug and combined mechanistic is difficult. As an example, our results have demonstrated that ODC_REMP_ treatment led to a significant increase in sphingolipids, whereas metformin and parthenolide are known to reduce lipid levels [78,79,97]. At the same time, there is minimal information on the impact on sphingolipids mediated by these drugs [77,98,99]. Simultaneously, these drugs do not solely influence metabolism, but cell signaling and the functionality of nuclear proteins. Our study offers the foundation to use the presented experimental platforms and combinations for further examinations. We aimed to develop, optimize and validate a drug combination to treat ccRCC, and in this context, we provided reliable data for further translation.

The cancer metabolism is known to be regulated by components of the tumor microenvironment, strongly influencing treatment efficacy [100]. Crosstalk between distinct cell populations protects the cancer cells or even promotes insensitivity to anti-cancer treatment [44]. Within the tumor microenvironment, the pH and the polarity can vary compared to the physiological values of the organ tissue. These alterations impact drug penetration and stability. Dose optimization may be considered once translated to 3D co-cultures, as would be performed for in vivo and in human translation [42].

In the presence of other cell lines, the expression of distinct receptors on the cell surface is altered, and through dose optimization, receptor saturation will be improved. To actively reduce the ATP levels in 3D co-cultures surrounded by a microenvironment including fibroblasts, endothelial cells and immune cell subsets, we chose to adjust the administration by repeated application, maintaining the same doses of the ODC_REMP_. There is consensus that it is beneficial to treat repeatedly at low doses than less frequently with high doses. This more complex and physiologically more relevant culture system was also used to visualize the selective targeting of cancer cells through ODC_REMP_ treatment by monitoring the presence of cell surface receptors. Notably, in the presence of immune cells, the ODC_REMP_ was slightly more active.

The comparison of the 3D co-cultures and ex vivo organoid-like cultures demonstrated a dissimilar reduction in the ATP levels after combination treatment. We hypothesize that the altered response relates to the tumor microenvironment, which differs significantly between the two culture types. The composition of the microenvironment of the ex vivo organoid-like cultures is more complex, involving human and murine cells interacting differently in ex vivo conditions from human cell lines co-cultured in vitro. The validation in ex vivo organoid-like cultures suggested that the ODC_REMP_ will be suitable for further translational studies. Additional in vivo studies will be conducted to determine the definite dosage of the drugs applied in combination and the most favorable schedule for translation, as well as the pharmacokinetics.

## 5. Conclusions

In summary, we demonstrated the effect and selectivity of an optimized multidrug combination for the treatment of sunitinib-resistant ccRCC cells in various in vitro systems. The strong impact of this combination on cell metabolism demonstrated an increase in lipids, particularly of the class of sphingolipids (lipids being vital for cell membranes were present to a greater extent after ODC treatment). Aligning to a recent study on drug combination optimization for the treatment of colorectal carcinoma [42], we consider continuing this line of investigation for ccRCC management for in vivo validation.

## 6. Patents

P.N.S. and M.R. are the inventors of the patent WO2021058587.

## Figures and Tables

**Table 1:** Drug–drug interactions and efficacy in cancer treatment.

Drug	Pharmaceutical Class	Indication	Targets	Clinical Stage	Potential Use in Cancer
Rapta-C	Chemotherapy; Anti-cancer [56]; Anti-angiogenic	-	p53-JNK pathway [33], protein; histones in DNA	preclinical	-
3-Hydroxy-tyraminium chloride	Anti-hypertension	Hemodynamic imbalance	System-wideagonist of dopamine type 1–5 receptors	approved	Glioma [57], colorectal cancer, tumor immunity ** [58]
Diclofenac sodium	Analgesics	Pain	Inhibition of prostaglandin synthesis by inhibition of the transiently expressed prostaglandin-endo-peroxide synthase-2 (PGES-2), also known as Cycloxygenase-2 (COX-2)	approved	Colorectal cancer, neuroblastoma, post-operative [59],pancreatic cancer ** [60]
Disulfiram	Acetaldehyde dehydrogenase inhibitor; Proteasome inhibitor	Alcoholism	Inhibits enzyme acetaldehyde dehydrogenase	approved	Any cancer type [61]
Erlotinib·HCl	Anti-cancer agent	Non-small cell lung cancer	Inhibition of the epidermal growth factor receptor	approved	
Famotidine	Anti-ulcer agent	Gastro-esophageal reflux disease	H2 Blocker and Histamine Receptor Antagonist	approved	Pre-operative, tumor immunity ** [62]
Haloprogin	Anti-infective agent; Anti-fungal agent	Skin infections (athlete’s foot, jock itch, ringworm, and tinea versicolor (a fungus)	GPCRs,dopamine and adrenergic receptors	discont.(side effects and available new drugs)	Melanoma [39],lung and pancreatic
Ivermectin	Anti-infective agent	Parasite infections	Glutamate-gated chloride channels central nervous system depression, and consequent ataxia, potentiation of inhibitory GABA-ergic synapses	approved	Cancer stem-like cells (breast), gastric cancer [63]
Metformin·HCl	Hypoglycemic agent	Diabetes type II	Decrease hepatic glucose production, mostly through a mild and transient inhibition of the mitochondrial respiratory chain complex I, the resulting decrease in hepatic energy status activates AMPK (AMP-activated protein kinase), a cellular metabolic sensor, providing a generally accepted mechanism for the action of metformin on hepatic gluconeogenesis	approved	Breast cancer stem cells [64], pancreatic cancer **, head and neck squamous cancer **
Parthenolide	Analgesics; Anti-inflammatory agent	HDAC1 inhibition; modulation of NF-kB-mediated inflammation	-	Under clinical investigation	(Breast) cancer stem(-like) cells, colorectal cancer [65,66]
Verapamil·HCl	Anti-hypertension	high blood pressure, chest pain from not enough blood flow to the heart muscle	Efflux pump inhibitor; voltage-dependent calcium channels	approved	Pancreatic tumor side population (stem-like) cells [67]
Acetylsalicylic acid	Analgesics;	Pain, fever	COX1 and COX2	approved	Gastrointestinal tract and lungs **, colorectal cancer ** [68]

** in clinical trials (https://www.cancer.gov/about-cancer/treatment/clinical-trials; accessed on 25 January 2021)

## Data Availability

Any underlying research materials related to this manuscript (for example data or models) can be requested by contacting the corresponding author.

## Supplementary Materials

The following are available online at https://www.mdpi.com/article/10.3390/cancers13163978/s1, Figure S1: Drug response curves for 11 repurposed drugs in sunitinib-naïve and -resistant ccRCC cell lines, Figure S2: Drug response curves for 11 repurposed drugs in non-cancerous cell lines, Figure S3: Drug response curves of Rapta-C in ccRCC and non-cancerous cell lines, Figure S4: TGMO screen in Caki-1 cells together with the therapeutic window, Figure S5: TGMO screen in Caki-1-SR clone 1 cells together with the therapeutic window, Figure S6: Calculation of the Combination Index for the ODCREMP, Figure S7: Linear regression models as statistical outputs in the modeling process to interpret the TGMO-based search, Figure S8: Validation of the anticancer efficacy of the ODCREMP in other ccRCC cell lines naïve or resistant to sunitinib, Figure S9: Activity of ODCREMP in non-cancerous cells, Figure S10: Activity of ODCREMP with a modified composition and dose together with its single drugs at different doses in non-cancerous cells, Figure S11: Global pathway analysis to extract information on the effect of the ODCREMP on cellular signaling and cellular mechanisms, Figure S12: Pathway analysis highlighting the cellular alterations after 24 h of ODCREMP treatment, Figure S13: Protein and RNA expression of CD41, CD44, HDAC, HIF-1α and ROCK1, Figure S14: Positive mode LC-HRMS/MS analysis of the drugs and their metabolites in the supernatant and cell extract of Caki-1-SR clone 1 cells, Figure S15: Ceramides after ODCREMP treatment, Figure S16: In silico prediction of ADME characteristics of the drugs contained in the ODCREMP, Figure S17: Analysis of the cell migration of Caki-1-SR clone 1-based 3D co-cultures in response to ODCREMP treatment, Figure S18: Adaptation of ODCREMP treatment schedule based on the readout of a single 72 h treatment and validation in sunitinib-resistant clones, Table S1: Information on selected drugs, Table S2: Selected drugs and doses from empirical and TGMO searches, Table S3: Drug–drug interactions between different drug classes, Table S4: Selection of 22 up- and downregulated genes comparing ODCREMP to CTRL applied for 24 h, Table S5: Network analysis ODCREMP 24 h to CTRL 24 h. FC ≤ −300 and ≥ 300, Table S6: RNA sequencing data accompanying protein expression results displayed in Supplementary Figure S13, Table S7: SMILES codes for drugs, Table S8: Top 15 predicted drug targets from Swiss Target Prediction, Table S9: Specification of lipids depicted in Figure 2E and Supplementary Figure S15, conducted with RefMet [76] hosted by Metabolomics Workbench, Table S10: In silico predicted pharmacokinetics using SwissADME, Table S11: Overview of drugs and stock solution preparation, Table S12: Fluorochrome-coupled antibodies for flow cytometry analysis.

## References

[1] Takebe T., Imai R., Ono S. (2018). The Current Status of Drug Discovery and Development as Originated in United States Academia: The Influence of Industrial and Academic Collaboration on Drug Discovery and Development. Clin. Transl. Sci..

[2] Talevi A., Bellera C.L. (2020). Challenges and opportunities with drug repurposing: Finding strategies to find alternative uses of therapeutics. Expert Opin. Drug Discov..

[3] Cha Y., Erez T., Reynolds I.J., Kumar D., Ross J., Koytiger G., Kusko R., Zeskind B., Risso S., Kagan E. (2018). Drug repurposing from the perspective of pharmaceutical companies. Br. J. Pharm..

[4] Langedijk J., Mantel-Teeuwisse A.K., Slijkerman D.S., Schutjens M.H. (2015). Drug repositioning and repurposing: Terminology and definitions in literature. Drug Discov. Today.

[5] Armando R.G., Mengual Gómez D.L., Gomez D.E. (2020). New drugs are not enough-drug repositioning in oncology: An update. Int. J. Oncol..

[6] Nowak-Sliwinska P., Scapozza L., Ruiz I., Altaba A. (2019). Drug repurposing in oncology: Compounds, pathways, phenotypes and computational approaches for colorectal cancer. Biochim. Biophys. Acta. Rev. Cancer.

[7] Sleire L., Forde H.E., Netland I.A., Leiss L., Skeie B.S., Enger P.O. (2017). Drug repurposing in cancer. Pharm. Res..

[8] Tannir N.M., Schwab G., Grünwald V. (2017). Cabozantinib: An Active Novel Multikinase Inhibitor in Renal Cell Carcinoma. Curr. Oncol. Rep..

[9] Xu J.X., Maher V.E., Zhang L., Tang S., Sridhara R., Ibrahim A., Kim G., Pazdur R. (2017). FDA Approval Summary: Nivolumab in Advanced Renal Cell Carcinoma After Anti-Angiogenic Therapy and Exploratory Predictive Biomarker Analysis. Oncologist.

[10] Gao X., McDermott D.F. (2018). Ipilimumab in combination with nivolumab for the treatment of renal cell carcinoma. Expert Opin. Biol..

[11] Caruso C. (2019). Checkpoint Inhibitor-TKI Combos Effective in RCC. Cancer Discov..

[12] Heckman-Stoddard B.M., DeCensi A., Sahasrabuddhe V.V., Ford L.G. (2017). Repurposing metformin for the prevention of cancer and cancer recurrence. Diabetologia.

[13] Hernandez J.J., Pryszlak M., Smith L., Yanchus C., Kurji N., Shahani V.M., Molinski S.V. (2017). Giving Drugs a Second Chance: Overcoming Regulatory and Financial Hurdles in Repurposing Approved Drugs As Cancer Therapeutics. Front. Oncol..

[14] Pantziarka P., Bouche G., Meheus L., Sukhatme V., Sukhatme V.P., Vikas P. (2014). The Repurposing Drugs in Oncology (ReDO) Project. Ecancermedicalscience.

[15] Verbaanderd C., Meheus L., Huys I., Pantziarka P. (2017). Repurposing Drugs in Oncology: Next Steps. Trends. Cancer.

[16] Zerbini L.F., Bhasin M.K., de Vasconcellos J.F., Paccez J.D., Gu X., Kung A.L., Libermann T.A. (2014). Computational repositioning and preclinical validation of pentamidine for renal cell cancer. Mol. Cancer.

[17] Jeong D.E., Song H.J.J., Lim S., Lee S.J.J., Lim J.E., Nam D.-H., Joo K.M., Jeong B.C., Jeon S.S., Choi H.Y. (2015). Repurposing the anti-malarial drug artesunate as a novel therapeutic agent for metastatic renal cell carcinoma due to its attenuation of tumor growth, metastasis, and angiogenesis. Oncotarget.

[18] Koudijs K.K.M., Terwisscha van Scheltinga A.G.T., Böhringer S., Schimmel K.J.M., Guchelaar H.-J. (2019). The impact of estimated tumour purity on gene expression-based drug repositioning of Clear Cell Renal Cell Carcinoma samples. Sci. Rep..

[19] Koudijs K.K.M., Terwisscha van Scheltinga A.G.T., Böhringer S., Schimmel K.J.M., Guchelaar H.-J. (2018). Personalised drug repositioning for Clear Cell Renal Cell Carcinoma using gene expression. Sci. Rep..

[20] Czarnecka A.M., Niedzwiedzka M., Porta C., Szczylik C. (2016). Hormone signaling pathways as treatment targets in renal cell cancer (Review). Int. J. Oncol..

[21] Song A., Zhang C., Meng X. (2021). Mechanism and application of metformin in kidney diseases: An update. Biomed. Pharmacother..

[22] Pasha M., Sivaraman S.K., Frantz R., Agouni A., Munusamy S. (2019). Metformin Induces Different Responses in Clear Cell Renal Cell Carcinoma Caki Cell Lines. Biomolecules.

[23] Yu H., Zhong X., Gao P., Shi J., Wu Z., Guo Z., Wang Z., Song Y. (2019). The Potential Effect of Metformin on Cancer: An Umbrella Review. Front. Endocrinol..

[24] Nowak-Sliwinska P., Weiss A., Ding X., Dyson P.J., van den Bergh H., Griffioen A.W., Ho C.M. (2016). Optimization of drug combinations using Feedback System Control. Nat. Protoc..

[25] Weiss A., Berndsen R.H., Ding X., Ho C.M., Dyson P.J., van den Bergh H., Griffioen A.W., Nowak-Sliwinska P. (2015). A streamlined search technology for identification of synergistic drug combinations. Sci. Rep..

[26] Lehar J., Krueger A.S., Zimmermann G.R., Borisy A.A. (2009). Therapeutic selectivity and the multi-node drug target. Discov. Med..

[27] Kummar S., Chen H.X., Wright J., Holbeck S., Millin M.D., Tomaszewski J., Zweibel J., Collins J., Doroshow J.H. (2010). Utilizing targeted cancer therapeutic agents in combination: Novel approaches and urgent requirements. Nat. Rev. Drug Discov..

[28] Fernandes Neto J.M., Nadal E., Ooft S.N., Bosdriesz E., Farre L., McLean C., Klarenbeek S., Jurgens A., Hagen H., Wang L. (2019). Multiple Low Dose (MLD) therapy: An effective strategy to treat EGFR inhibitor-resistant NSCLC tumours. bioRxiv.

[29] Motzer R.J., Tannir N.M., McDermott D.F., Aren Frontera O., Melichar B., Choueiri T.K., Plimack E.R., Barthelemy P., Porta C., George S. (2018). Nivolumab plus Ipilimumab versus Sunitinib in Advanced Renal-Cell Carcinoma. N. Engl. J. Med..

[30] McDermott D.F., Huseni M.A., Atkins M.B., Motzer R.J., Rini B.I., Escudier B., Fong L., Joseph R.W., Pal S.K., Reeves J.A. (2018). Clinical activity and molecular correlates of response to atezolizumab alone or in combination with bevacizumab versus sunitinib in renal cell carcinoma. Nat. Med..

[31] Xue H., Li J., Xie H., Wang Y. (2018). Review of Drug Repositioning Approaches and Resources. Int. J. Biol. Sci..

[32] Setoain J., Franch M., Martinez M., Tabas-Madrid D., Sorzano C.O., Bakker A., Gonzalez-Couto E., Elvira J., Pascual-Montano A. (2015). NFFinder: An online bioinformatics tool for searching similar transcriptomics experiments in the context of drug repositioning. Nucleic Acids Res..

[33] Chatterjee S., Kundu S., Bhattacharyya A., Hartinger C.G., Dyson P.J. (2008). The ruthenium(II)-arene compound RAPTA-C induces apoptosis in EAC cells through mitochondrial and p53-JNK pathways. J. Biol. Inorg. Chem..

[34] Scolaro C., Hartinger C.G., Allardyce C.S., Keppler B.K., Dyson P.J. (2008). Hydrolysis study of the bifunctional antitumour compound RAPTA-C, [Ru(eta6-p-cymene)Cl2(pta)]. J. Inorg. Biochem..

[35] Berndsen R.H., Weiss A., Abdul U.K., Wong T.J., Meraldi P., Griffioen A.W., Dyson P.J., Nowak-Sliwinska P. (2017). Combination of ruthenium(II)-arene complex [Ru(eta(6)-p-cymene)Cl2(pta)] (RAPTA-C) and the epidermal growth factor receptor inhibitor erlotinib results in efficient angiostatic and antitumor activity. Sci. Rep..

[36] Kilpin K.J., Cammack S.M., Clavel C.M., Dyson P.J. (2013). Ruthenium(II) arene PTA (RAPTA) complexes: Impact of enantiomerically pure chiral ligands. Dalton. Trans..

[37] Nowak-Sliwinska P., van Beijnum J.R., Casini A., Nazarov A.A., Wagnieres G., van den Bergh H., Dyson P.J., Griffioen A.W. (2011). Organometallic ruthenium(II) arene compounds with antiangiogenic activity. J. Med. Chem..

[38] Rausch M., Dyson P.J., Nowak-Sliwinska P. (2019). Recent Considerations in the Application of RAPTA-C for Cancer Treatment and Perspectives for Its Combination with Immunotherapies. Adv. Ther..

[39] Riedel T., Demaria O., Zava O., Joncic A., Gilliet M., Dyson P.J. (2018). Drug Repurposing Approach Identifies a Synergistic Drug Combination of an Antifungal Agent and an Experimental Organometallic Drug for Melanoma Treatment. Mol. Pharm..

[40] Rausch M., Weiss A., Achkhanian J., Rotari A., Nowak-Sliwinska P. (2020). Identification of low-dose multidrug combinations for sunitinib-naive and pre-treated renal cell carcinoma. Br. J. Cancer.

[41] Rausch M., Weiss A., Zoetemelk M., Piersma S.R., Jimenez C.R., van Beijnum J.R., Nowak-Sliwinska P. (2020). Optimized Combination of HDACI and TKI Efficiently Inhibits Metabolic Activity in Renal Cell Carcinoma and Overcomes Sunitinib Resistance. Cancers.

[42] Zoetemelk M., Ramzy G.M., Rausch M., Koessler T., van Beijnum J.R., Weiss A., Mieville V., Piersma S.R., de Haas R.R., Delucinge-Vivier C. (2020). Optimized low-dose combinatorial drug treatment boosts selectivity and efficacy of colorectal carcinoma treatment. Mol. Oncol..

[43] Allardyce C.S., Dyson P.J., Ellis D.J., Heath S.L. (2001). [Ru(η--cymene)Cl(pta)] (pta = 1,3,5-triaza-7-phosphatricyclo- [3.3.1.1]decane): A water soluble compound that exhibits pH dependent DNA binding providing selectivity for diseased cells. Chem. Commun..

[44] Rausch M., Blanc L., Silva O.D.S., Dormond O., Griffioen A.W., Nowak-Sliwinska P. (2021). Characterization of Renal Cell Carcinoma Heterotypic 3D Co-Cultures with Immune Cell Subsets. Cancers.

[45] Dobin A., Davis C.A., Schlesinger F., Drenkow J., Zaleski C., Jha S., Batut P., Chaisson M., Gingeras T.R. (2013). STAR: Ultrafast universal RNA-seq aligner. Bioinformatics.

[46] Robinson M.D., McCarthy D.J., Smyth G.K. (2010). edgeR: A Bioconductor package for differential expression analysis of digital gene expression data. Bioinformatics.

[47] Enrichr. http://amp.pharm.mssm.edu/Enrichr.

[48] Gene Expression Omnibus. www.ncbi.nlm.nih.gov/geo.

[49] Clarivate. https://clarivate.com/.

[50] Rausch M., Rutz A., Allard P.M., Delucinge-Vivier C., Docquier M., Dormond O., Wolfender J.L., Nowak-Sliwinska P. (2021). Molecular and functional analysis of sunitinib resistance induction in human renal cell carcinoma cells. Int. J. Mol. Sci..

[51] Chambers M.C., Maclean B., Burke R., Amodei D., Ruderman D.L., Neumann S., Gatto L., Fischer B., Pratt B., Egertson J. (2012). A cross-platform toolkit for mass spectrometry and proteomics. Nat. Biotechnol..

[52] Pluskal T., Castillo S., Villar-Briones A., Oresic M. (2010). MZmine 2: Modular framework for processing, visualizing, and analyzing mass spectrometry-based molecular profile data. BMC Bioinform..

[53] Kondo J., Ekawa T., Endo H., Yamazaki K., Tanaka N., Kukita Y., Okuyama H., Okami J., Imamura F., Ohue M. (2019). High-throughput screening in colorectal cancer tissue-originated spheroids. Cancer Sci..

[54] Zeng L., Gupta P., Chen Y., Wang E., Ji L., Chao H., Chen Z.-S. (2017). The development of anticancer ruthenium(ii) complexes: From single molecule compounds to nanomaterials. Chem. Soc. Rev..

[55] Lee S.Y., Kim C.Y., Nam T.-G. (2020). Ruthenium Complexes as Anticancer Agents: A Brief History and Perspectives. Drug Des. Dev. Ther..

[56] Babak M.V., Meier S.M., Huber K.V.M., Reynisson J., Legin A.A., Jakupec M.A., Roller A., Stukalov A., Gridling M., Bennett K.L. (2015). Target profiling of an antimetastatic RAPTA agent by chemical proteomics: Relevance to the mode of action. Chem. Sci..

[57] Lan Y.L., Wang X., Xing J.S., Yu Z.L., Lou J.C., Ma X.C., Zhang B. (2017). Anti-cancer effects of dopamine in human glioma: Involvement of mitochondrial apoptotic and anti-inflammatory pathways. Oncotarget.

[58] Zhang X., Liu Q., Liao Q., Zhao Y. (2017). Potential Roles of Peripheral Dopamine in Tumor Immunity. J. Cancer.

[59] Pantziarka P., Sukhatme V., Bouche G., Meheus L., Sukhatme V.P. (2016). Repurposing Drugs in Oncology (ReDO)-diclofenac as an anti-cancer agent. Ecancermedicalscience.

[60] Breuer S., Maimon O., Appelbaum L., Peretz T., Hubert A. (2013). TL-118-anti-angiogenic treatment in pancreatic cancer: A case report. Med. Oncol..

[61] Ding N., Zhu Q. (2018). Disulfiram combats cancer via crippling valosin-containing protein/p97 segregase adaptor NPL4. Transl. Cancer Res..

[62] Parshad R., Kapoor S., Gupta S.D., Kumar A., Chattopadhyaya T.K. (2002). Does famotidine enhance tumor infiltrating lymphocytes in breast cancer? Results of a randomized prospective pilot study. Acta. Oncol..

[63] Nambara S., Masuda T., Nishio M., Kuramitsu S., Tobo T., Ogawa Y., Hu Q., Iguchi T., Kuroda Y., Ito S. (2017). Antitumor effects of the antiparasitic agent ivermectin via inhibition of Yes-associated protein 1 expression in gastric cancer. Oncotarget.

[64] Zi F., Zi H., Li Y., He J., Shi Q., Cai Z. (2018). Metformin and cancer: An existing drug for cancer prevention and therapy. Oncol. Lett..

[65] Liu Y.C., Kim S.L., Park Y.R., Lee S.-T., Kim S.W. (2017). Parthenolide promotes apoptotic cell death and inhibits the migration and invasion of SW620 cells. Intest. Res..

[66] Ghantous A., Sinjab A., Herceg Z., Darwiche N. (2013). Parthenolide: From plant shoots to cancer roots. Drug Discov. Today.

[67] Zhao L., Zhao Y., Schwarz B., Mysliwietz J., Hartig R., Camaj P., Bao Q., Jauch K.W., Guba M., Ellwart J.W. (2016). Verapamil inhibits tumor progression of chemotherapy-resistant pancreatic cancer side population cells. Int. J. Oncol..

[68] Elwood P.C., Morgan G., Pickering J.E., Galante J., Weightman A.L., Morris D., Kelson M., Dolwani S. (2016). Aspirin in the Treatment of Cancer: Reductions in Metastatic Spread and in Mortality: A Systematic Review and Meta-Analyses of Published Studies. PLoS ONE.

[69] Cavalla D. (2019). Using human experience to identify drug repurposing opportunities: Theory and practice. Br. J. Clin. Pharmacol..

[70] Pushpakom S., Iorio F., Eyers P.A., Escott K.J., Hopper S., Wells A., Doig A., Guilliams T., Latimer J., McNamee C. (2018). Drug repurposing: Progress, challenges and recommendations. Nat. Rev. Drug. Discov..

[71] Weiss A., Le Roux-Bourdieu M., Zoetemelk M., Ramzy G.M., Rausch M., Harry D., Miljkovic-Licina M., Falamaki K., Wehrle-Haller B., Meraldi P. (2019). Identification of a Synergistic Multi-Drug Combination Active in Cancer Cells via the Prevention of Spindle Pole Clustering. Cancers.

[72] Chou T.C. (2010). Drug combination studies and their synergy quantification using the Chou-Talalay method. Cancer Res..

[73] Daina A., Michielin O., Zoete V. (2019). SwissTargetPrediction: Updated data and new features for efficient prediction of protein targets of small molecules. Nucleic. Acids. Res..

[74] Artner C., Holtkamp H.U., Hartinger C.G., Meier-Menches S.M. (2017). Characterizing activation mechanisms and binding preferences of ruthenium metallo-prodrugs by a competitive binding assay. J. Inorg. Biochem..

[75] Holtkamp H.U., Movassaghi S., Morrow S.J., Kubanik M., Hartinger C.G. (2018). Metallomic study on the metabolism of RAPTA-C and cisplatin in cell culture medium and its impact on cell accumulation. Metallomics.

[76] Fahy E., Subramaniam S. (2020). RefMet: A reference nomenclature for metabolomics. Nat. Methods.

[77] Pradas I., Rovira-Llopis S., Naudí A., Bañuls C., Rocha M., Hernandez-Mijares A., Pamplona R., Victor V.M., Jové M. (2019). Metformin induces lipid changes on sphingolipid species and oxidized lipids in polycystic ovary syndrome women. Sci. Rep..

[78] Van Stee M.F., de Graaf A.A., Groen A.K. (2018). Actions of metformin and statins on lipid and glucose metabolism and possible benefit of combination therapy. Cardiovasc. Diabetol..

[79] Kim C.Y., Kang B., Hong J., Choi H.-S. (2019). Parthenolide inhibits lipid accumulation via activation of Nrf2/Keap1 signaling during adipocyte differentiation. Food Sci. Biotechnol..

[80] Posocco B., Buzzo M., Giodini L., Crotti S., D’Aronco S., Traldi P., Agostini M., Marangon E., Toffoli G. (2018). Analytical aspects of sunitinib and its geometric isomerism towards therapeutic drug monitoring in clinical routine. J. Pharm. Biomed. Anal..

[81] Liberti M.V., Locasale J.W. (2016). The Warburg Effect: How Does it Benefit Cancer Cells?. Trends. Biochem. Sci..

[82] Vander Heiden M.G., Cantley L.C., Thompson C.B. (2009). Understanding the Warburg effect: The metabolic requirements of cell proliferation. Science.

[83] Daina A., Michielin O., Zoete V. (2017). SwissADME: A free web tool to evaluate pharmacokinetics, drug-likeness and medicinal chemistry friendliness of small molecules. Sci. Rep..

[84] Daina A., Zoete V. (2016). A BOILED-Egg To Predict Gastrointestinal Absorption and Brain Penetration of Small Molecules. ChemMedChem.

[85] Huinen Z.R., Huijbers E.J.M., van Beijnum J.R., Nowak-Sliwinska P., Griffioen A.W. (2021). Anti-angiogenic agents—overcoming tumour endothelial cell anergy and improving immunotherapy outcomes. Nat. Rev. Clin. Oncol..

[86] Bayat Mokhtari R., Homayouni T.S., Baluch N., Morgatskaya E., Kumar S., Das B., Yeger H. (2017). Combination therapy in combating cancer. Oncotarget.

[87] Zhang Z., Zhou L., Xie N., Nice E.C., Zhang T., Cui Y., Huang C. (2020). Overcoming cancer therapeutic bottleneck by drug repurposing. Signal Transduct. Target. Ther..

[88] Suissa S., Azoulay L. (2014). Metformin and Cancer: Mounting Evidence Against an Association. Diabetes Care.

[89] Malek M., Aghili R., Emami Z., Khamseh M.E. (2013). Risk of Cancer in Diabetes: The Effect of Metformin. ISRN Endocrinol..

[90] Ugwueze C.V., Ogamba O.J., Young E.E., Onyenekwe B.M., Ezeokpo B.C. (2020). Metformin: A Possible Option in Cancer Chemotherapy. Anal. Cell. Pathol..

[91] Richard S.M., Martinez Marignac V.L. (2015). Sensitization to oxaliplatin in HCT116 and HT29 cell lines by metformin and ribavirin and differences in response to mitochondrial glutaminase inhibition. J. Cancer Res..

[92] Sun L., Yuan W., Wen G., Yu B., Xu F., Gan X., Tang J., Zeng Q., Zhu L., Chen C. (2020). Parthenolide inhibits human lung cancer cell growth by modulating the IGF-1R/PI3K/Akt signaling pathway. Oncol. Rep..

[93] Weiss A., Ding X., van Beijnum J.R., Wong I., Wong T.J., Berndsen R.H., Dormond O., Dallinga M., Shen L., Schlingemann R.O. (2015). Rapid optimization of drug combinations for the optimal angiostatic treatment of cancer. Angiogenesis.

[94] Ratanaphan A., Temboot P., Dyson P.J. (2010). In vitro ruthenation of human breast cancer suppressor gene 1 (BRCA1) by the antimetastasis compound RAPTA-C and its analogue CarboRAPTA-C. Chem. Biodivers..

[95] Jafari N., Nazeri S., Enferadi S.T. (2018). Parthenolide reduces metastasis by inhibition of vimentin expression and induces apoptosis by suppression elongation factor α − 1 expression. Phytomedicine.

[96] Makhov P., Naito S., Haifler M., Kutikov A., Boumber Y., Uzzo R.G., Kolenko V.M. (2018). The convergent roles of NF-κB and ER stress in sunitinib-mediated expression of pro-tumorigenic cytokines and refractory phenotype in renal cell carcinoma. Cell Death Dis..

[97] Yuan L., Wang Z., Zhang D., Wang J. (2020). Metabonomic study of the intervention effects of Parthenolide on anti-thyroid cancer activity. J. Chromatogr. B.

[98] Meacham W.D., Antoon J.W., Burow M.E., Struckhoff A.P., Beckman B.S. (2009). Sphingolipids as Determinants of Apoptosis and Chemoresistance in the MCF-7 Cell Model System. Exp. Biol. Med..

[99] Lewis A.C., Wallington-Beddoe C.T., Powell J.A., Pitson S.M. (2018). Targeting sphingolipid metabolism as an approach for combination therapies in haematological malignancies. Cell Death Discov..

[100] Cairns R.A., Harris I.S., Mak T.W. (2011). Regulation of cancer cell metabolism. Nat. Rev. Cancer.

