# Supplementary material for: Drug Repurposing to Identify a Synergistic High-Order Drug Combination to Treat Sunitinib-Resistant Renal Cell Carcinoma
Source: Cancers (Basel). 2021 Aug 6;13(16):3978. doi: 10.3390/cancers13163978 (PMC8391235; doi:10.3390/cancers13163978)
Supplement: Supplementary file 1 [file cancers-13-03978-s001.zip › cancers-1308081-supplementary.pdf]

Supplementary Materials: Drug Repurposing to Indentify a Synergistic High-Order Drug Combination to Treat Sunitinib-Resistant Renal Cell Carcinoma

Magdalena Rausch, Adriano Rutz, Pierre-Marie Allard, Céline Delucinge-Vivier, Mylène Docquier, Olivier Dormond, Paul J. Dyson, Jean-Luc Wolfender, Patrycja Nowak-Sliwinska

1. Supplementary Information S1

Data analysis of mass spectrometry data

The manually curated peaklist was treated with MetaboDrift [1] using the LOESS method. Statistical and functional analysis were performed with MetaboAnalyst [2]. Functional analysis was done using lipids (main class) and MFN databases. Allowed adducts were: "M [1+]","M+H [1+]","M+2H [2+]","M+3H [3+]","M+Na [1+]","M+H+Na [2+]","M-H2O+H [1+]","M-H4O2+H [1+]","M(C13)+H [1+]","M(C13)+2H [2+]","M(C13)+3H [3+]","M(S34)+H [1+]","M(Cl37)+H [1+]","M-NH3+H [1+]","M-CO+H [1+]","M-CO2+H [1+]","M-HCOOH+H [1+]","M-C3H4O2+H [1+]","M+2Na [2+]","M+3Na [3+]","M+H+2Na [3+]","M+2H+Na [3+]","M+2Na-H [1+]","M+ACN+H [1+]","M+ACN+2H [2+]","M+ACN+Na [1+]","M+2ACN+2H [2+]","M+3ACN+2H [2+]","M+2ACN+H [1+]","M+H+NH4 [2+]","M+NH4 [1+]","2M+H [1+]","2M+NH4 [1+]","2M+Na [1+]","2M+ACN+H [1+]","2M+ACN+Na [1+]", and "M+HCOONa [1+]". When multiple annotation classes were given, the most represented one was chosen.

We further provided an online visualization of our data accessible for anyone: https://gnps-lcms.ucsd.edu/?xic_mz=659.2850&xic_formula=&xic_peptide=&xic_tolerance=0.5&xic_ppm_tolerance=10&xic_tolerance_unit=ppm&xic_rt_window=&xic_norm=False&xic_file_grouping=MZ&xic_integration_type=AUC&show_ms2_markers=True&ms2marker_color=blue&ms2marker_size=5&ms2_identifier=None&show_lcms_2nd_map=False&map_plot_zoom=%7b%7d&polarity_filtering=None&polarity_filtering2=None&tic_option=BPI&overlay_usi=None&overlay_mz=row+m/z&overlay_rt=row+retention+time&overlay_color=&overlay_size=&overlay_hover=&overlay_filter_column=&overlay_filter_value=&feature_finding_type=Off&feature_finding_ppm=10&feature_finding_noise=10000&feature_finding_min_peak_rt=0.05&feature_finding_max_peak_rt=1.5&feature_finding_rt_tolerance=0.3&sychronization_session_id=c0e1e23286b348368229b6a580dee1a1&chromatogram_options=%5b%5d&comment=&map_plot_color_scale=Hot_r&map_plot_quantization_level=Medium&plot_theme=plotly_white#{usi:%20mzspec:MSV000087459:peak/20210209_ctr_1_pos.mzML\nmzspec:MSV000087459:peak/20210209_ctr_2_pos.mzML\nmzspec:MSV000087459:peak/20210209_ctr_3_pos.mzML\nmzspec:MSV000087459:peak/20210209_odc_1_pos.mzML\nmzspec:MSV000087459:peak/20210209_odc_2_pos.mzML\nmzspec:MSV000087459:peak/20210209_odc_3_pos.mzML\nmzspec:MSV000087459:peak/20210209_qc_1_pos.mzML\nmzspec:MSV000087459:peak/20210209_qc_2_pos.mzML\nmzspec:MSV000087459:peak/20210209_qc_3_pos.mzML\nmzspec:MSV000087459:peak/20210209_qc_4_pos.mzML,%20usi2:%20mzspec:MSV000087459:peak/20210209_ctr_1_pos.mzML\nmzspec:MSV000087459:peak/20210209_ctr_2_pos.mzML\nmzspec:MSV000087459:peak/20210209_ctr_3_pos.mzML\nmzspec:MSV000087459:peak/20210209_odc_1_pos.mzML\nmzspec:MSV000087459:peak/20210209_odc_2_pos.mzML\nmzspec:MSV000087459:peak/20210209_odc_3_pos.mzML\nmzspec:MSV000087459:peak/20210209_qc_1_pos.mzML\nmz-

spec:MSV000087459:peak/20210209_qc_2_pos.mzML\nmz-spec:MSV000087459:peak/20210209_qc_3_pos.mzML\nmz-spec:MSV000087459:peak/20210209_qc_4_pos.mzML}

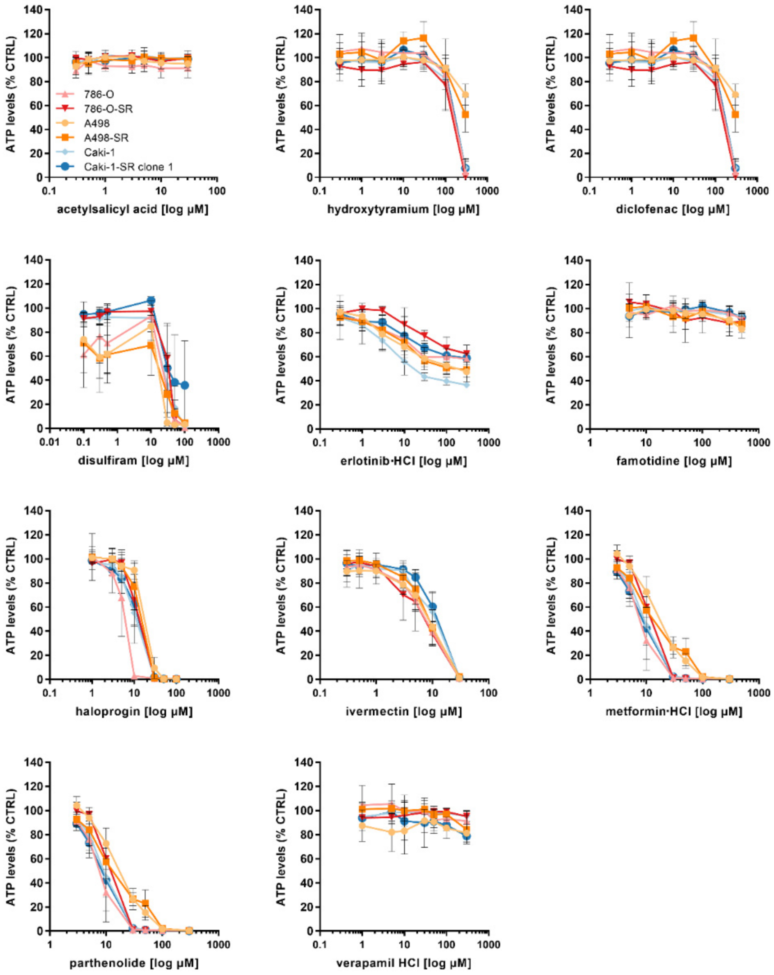

Figure S1. Drug response curves for 11 repurposed drugs in sunitinib-naïve and -resistant ccRCC cell lines. Drug dose-response curves were performed in A498, Caki-1, 786-O and the same cell lines resistant to sunitinib (-SR). Error bars represent the standard deviation of N = 3–5 independent experiments.

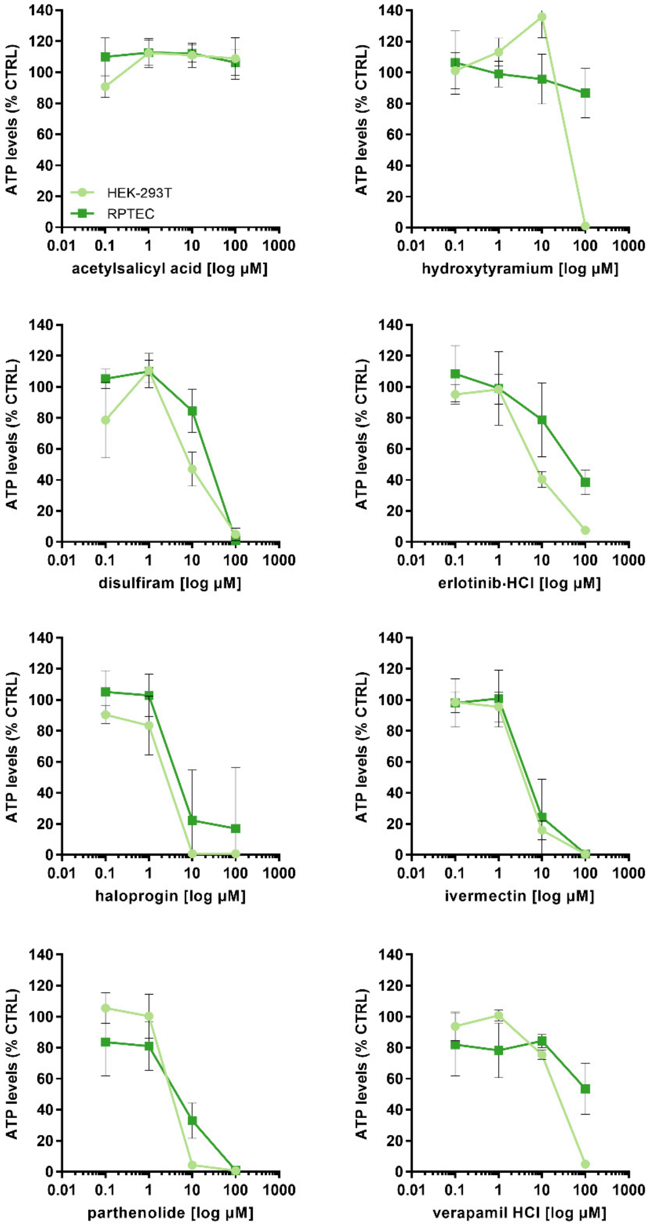

Figure S2. Drug response curves for 11 repurposed drugs in non-cancerous cell lines. The sensitivity to increasing doses of the 11 repurposed drugs described was evaluated in non-cancerous cell lines HEK-293T and RPTEC cells. Error bars represent the standard deviation of N = 3–5 independent experiments.

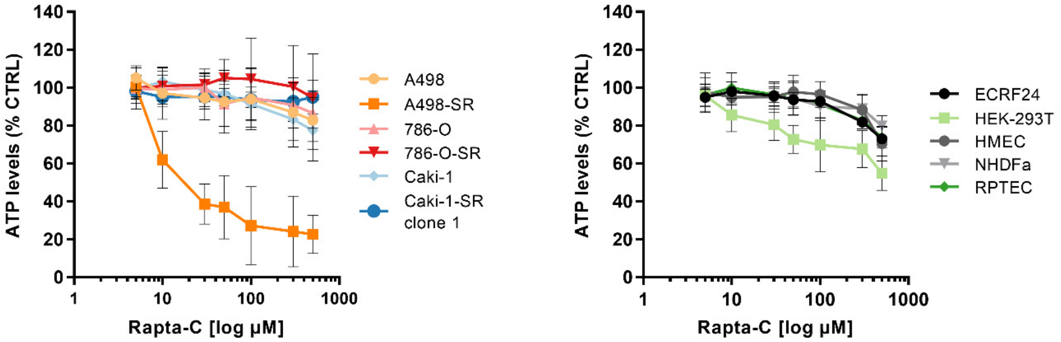

Figure S3. Drug response curves of Rapta-C in ccRCC and non-cancerous cell lines. The response of sunitinib-naïve, sunitinib-resistant ccRCC cell lines as well as non-cancerous cell lines to Rapta-C was measured monitoring the extra- and intracellular ATP levels. In addition to HEK-293T and RPTEC cells, the drug activity was also evaluated in ECRF24, HMEC and NHDFα cells. Error bars represent the standard deviation of $N = 3$–5 independent experiments.

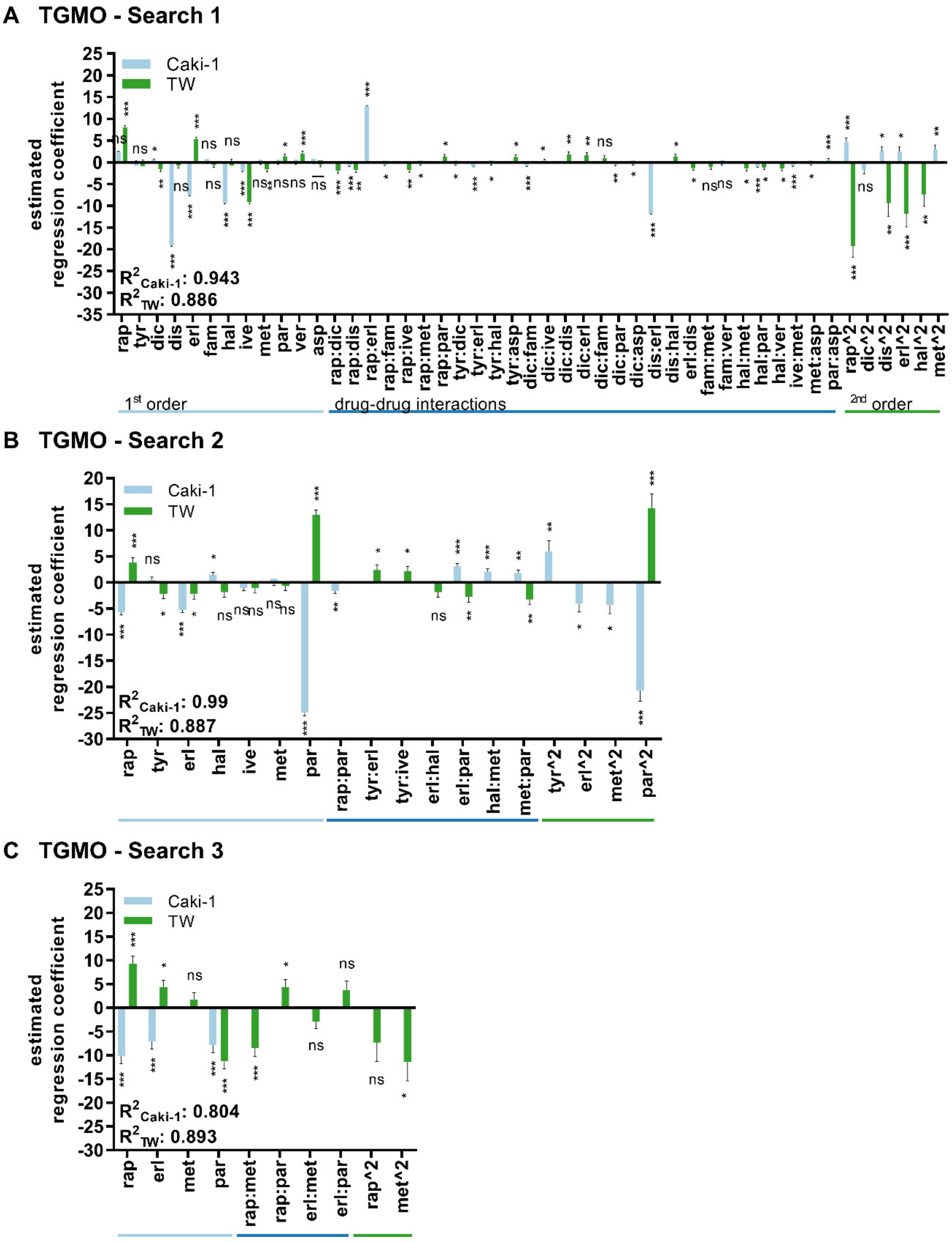

Figure S4. TGMO screen in Caki-1 cells together with the therapeutic window. Computational modeling based on experimental data input calculating estimated regression coefficients describing drug parameters such as single drug 1st order (light blue), drug-drug interactions (dark blue) and single drug 2nd order (green) activity. The data shows the screen performed in Caki-1 cells (light blue bars) and non-malignant RPTEC cells. As a result a therapeutic window is generated (green bars). (**A**) Search 1, in which 155 drug combinations with 12 drugs were screened (*N* = 2). (**B**) Search 2, in which 50 drug combinations with 7 drugs were screened (*N* = 2). (**C**) Search 3, screening of 25 drug combinations with 4 drugs (*N* = 2). Error bars represent the standard deviation and significance of estimated regression coefficients was determined with a one-way ANOVA; * $p < 0.05$, ** $p < 0.01$ and *** $p < 0.001$. R^2 represents model accuracy in a coefficient of multiple determination.

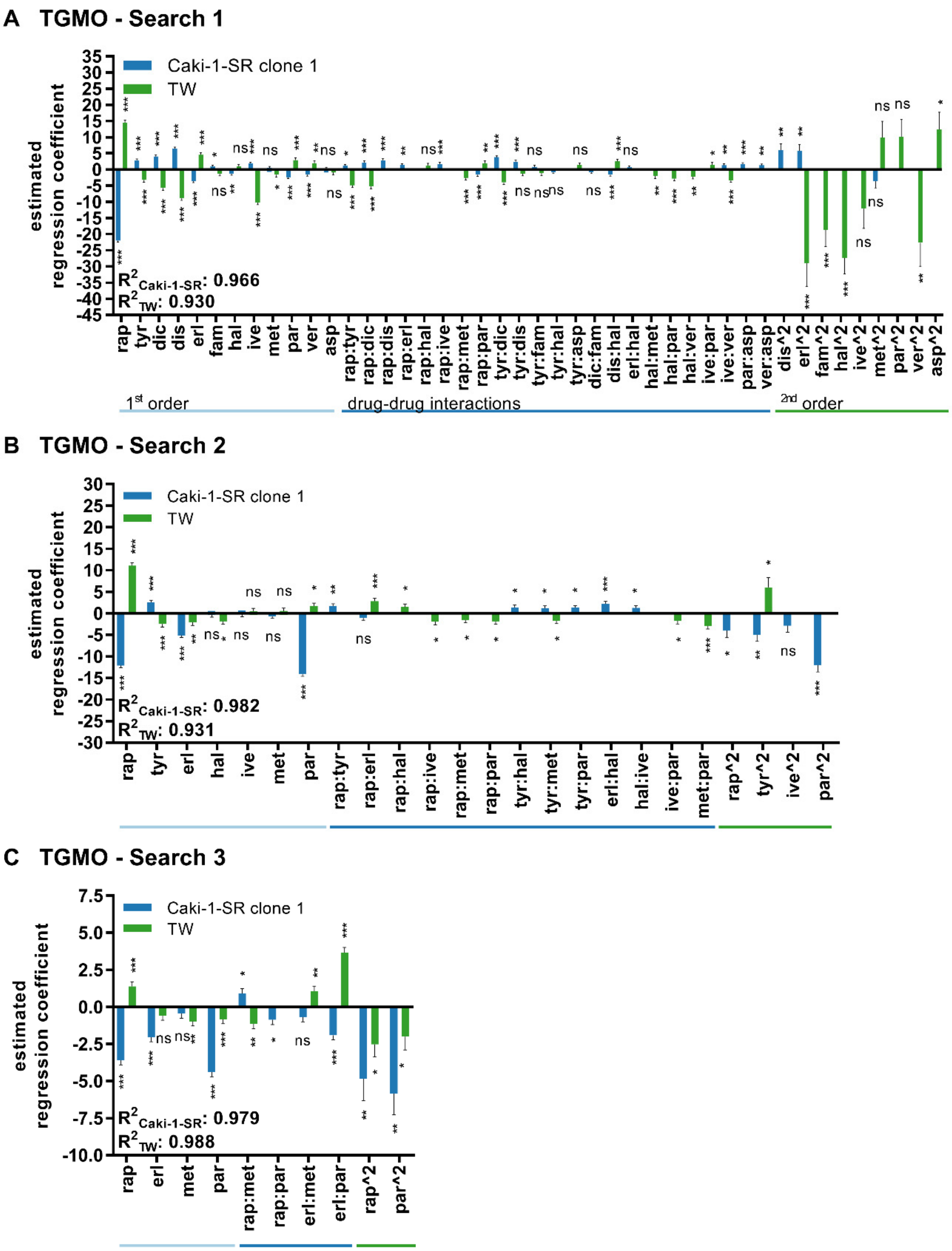

Figure S5. TGMO screen in Caki-1-SR clone 1 cells together with the therapeutic window. Computational modeling based on experimental data input calculating estimated regression coefficients describing drug parameters such as single drug 1st order, drug-drug interactions and single drug 2nd order activity. The data shows the screen performed in Caki-1-SR clone 1 cells (blue bars) and non-malignant RPTEC cells. As a result a therapeutic window is generated (green bars). (**A**) Iteration 1, in which 155 drug combinations with 12 drugs were screened ($N = 2$). (**B**) Iteration 2, in which 50 drug combinations with 7 drugs were screened ($N = 2$). (**C**) Iteration 3, screening of 25 drug combinations with 4 drugs ($N = 2$). Error bars represent the standard deviation and significance of estimated regression coefficients was determined with a one-way ANOVA; * $p < 0.05$, ** $p < 0.01$ and *** $p < 0.001$. R2 represents model accuracy in a coefficient of multiple determination.

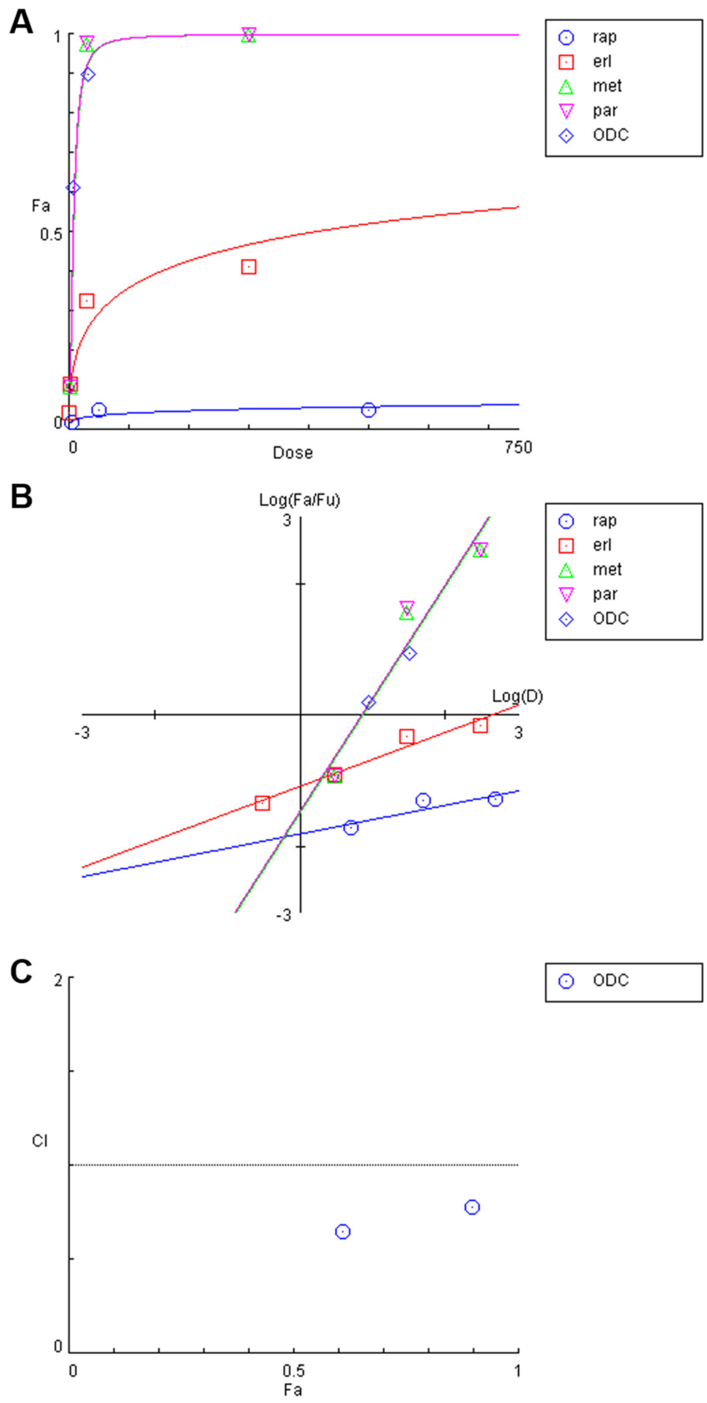

Figure S6. Calculation of the Combination Index for the ODCREMP. Isobolograms representing the (**A**) dose- and (**B**) median effect of Rapta-C (rap), erlotinib (erl), metformin (met) and parthenolide (par) together with the four-drug combination (ODC(REMP)) in Caki-1-SR clone 1 cells. (**C**) The Combination Index (CI) was calculated for the four-drug combination at two different doses and is presented in the combination index blot.

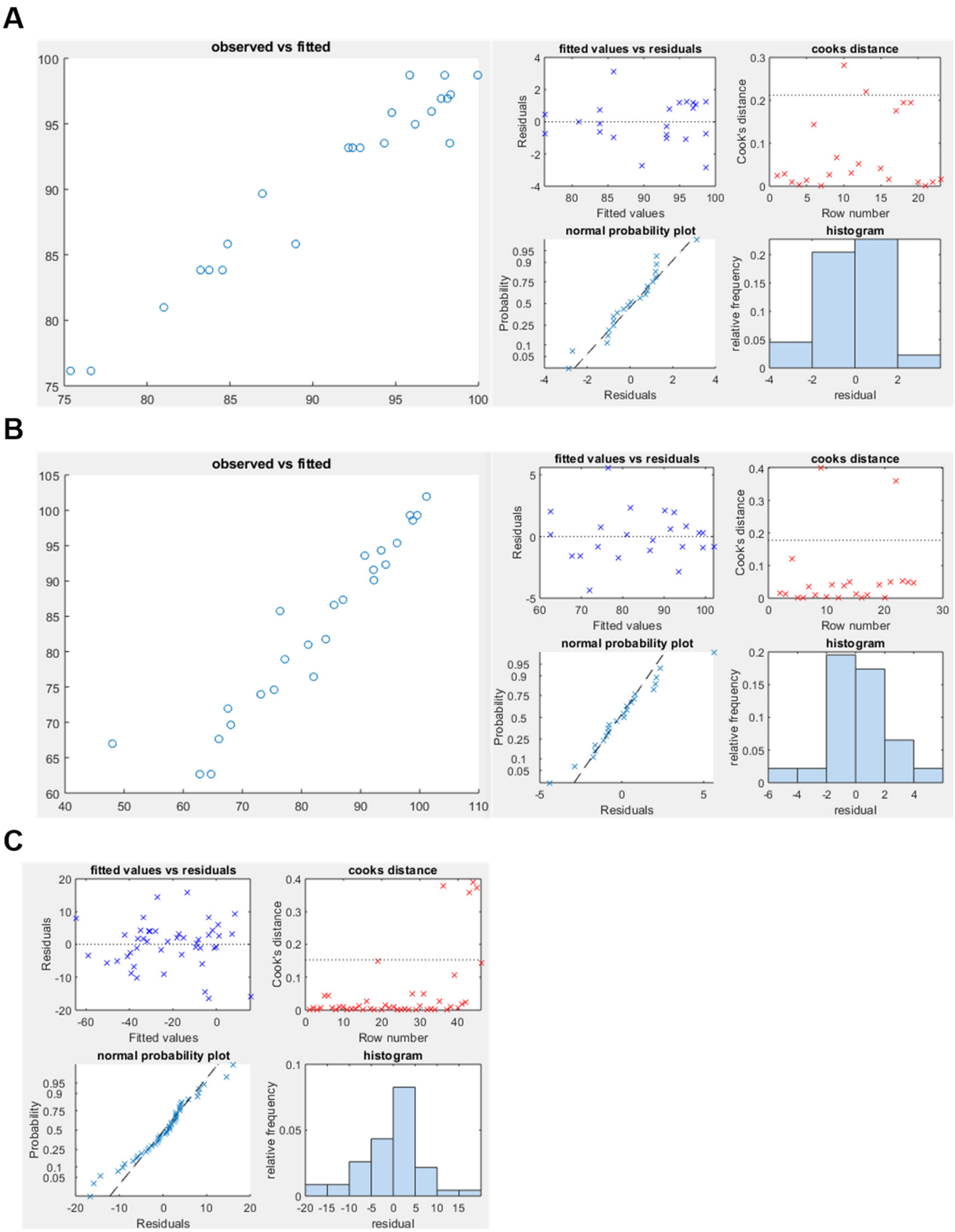

Figure S7. Linear regression models as statistical output in the modeling process to interpret the TGMO-based search. Assessment of the accuracy and predictive value of the models through accompanying model analysis. The model analysis of Search 3, performed in (**A**) Caki-1-SR clone 1 cells, (**B**) RPTEC cells and (**C**) the therapeutic window. Observed vs. fitted values plot with the multiple determination (R^2) (left plot), residual analysis plot of data to visualize constant variance (top left), Cook's distance plot (top right), Q-Q plot (bottom left) and histogram of residuals (bottom right).

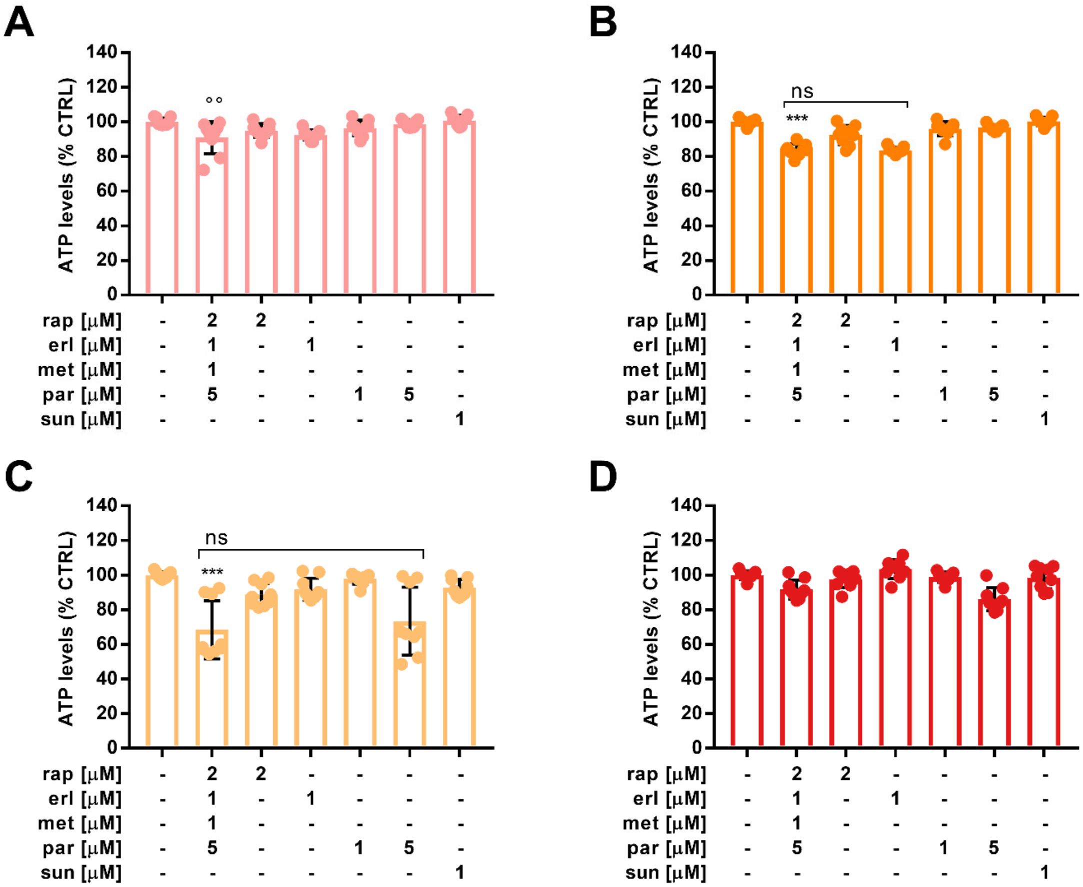

Figure S8. Validation of the anticancer efficacy of the ODC$_{\mathrm{REMP}}$ in other ccRCC cell lines naïve or resistant to sunitinib. Bar graphs demonstrating the cellular response measured as ATP levels to ODC$_{\mathrm{REMP}}$ and monotherapy treatment in (**A**) A498, (**B**) A498-SR, (**C**) 786-O and (**D**) 786-O-SR cells. Error bars represent the standard deviation and significance was calculated of $N = 3$ independent experiments using a one-way ANOVA test; ** $p < 0.01$, *** $p < 0.001$ represents the significance versus the sham-control.

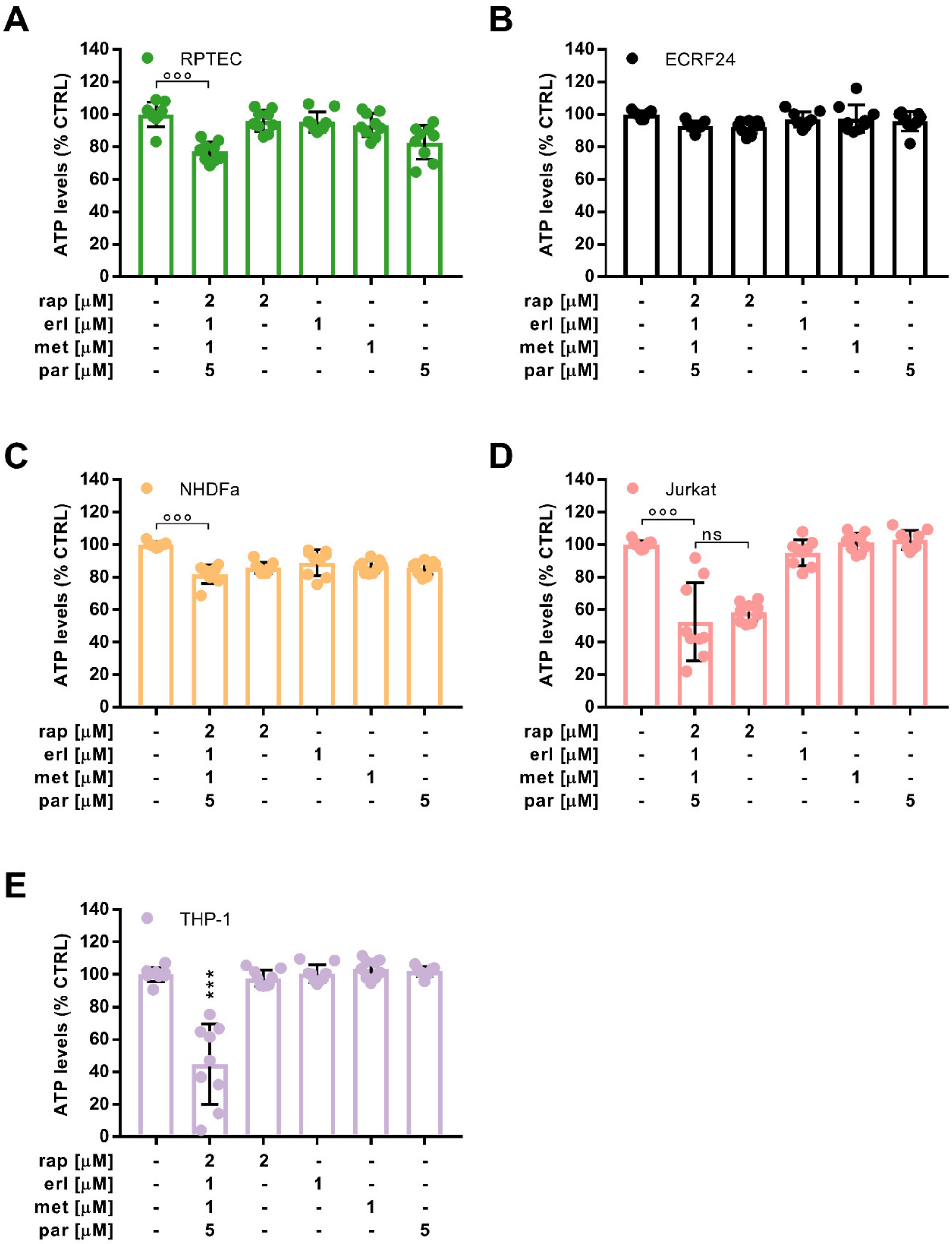

Figure S9. Activity of ODCREMP in non-cancerous cells. Assesment of the ODCREMP and its monotherapies in non-cancerous (**A**) PRTEC, (**B**) ECRF24, (**C**) NHDFα, (**D**) Jurkat and (**E**) THP-1 cells evaluating the level of ATP after 72 hours of treatment in comparison to the sham-control (CTRL). These cells have been chosen because they can be found in the kidney tissue. Error bars represent the standard deviation and significance was calculated of $N = 3$ independent experiments using a one-way ANOVA test; *** $p < 0.001$ represents the significance versus the sham-control.

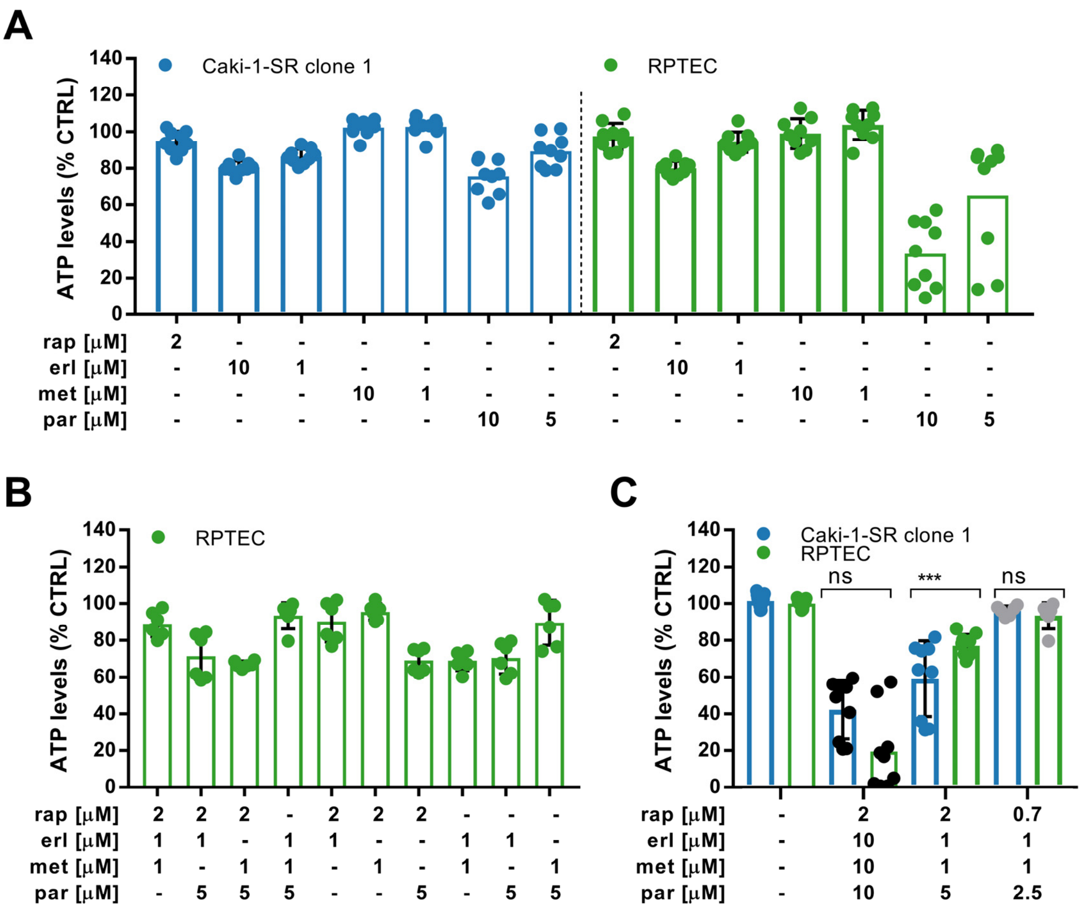

Figure S10. Activity of ODCREMP modified in its composition and dose together with its single drugs at different doses in non-cancerous cells. (**A**) The efficacy on the ATP production of the monotherapies at different dose levels measured in Caki-1-SR clone 1 and RPTEC cells after 72 hour treatment. Error bars represent the standard deviation and significance was calculated of $N = 3$ independent experiments. (**B**) Evaluation of three- and two-drug combinations in RPTEC cells. Error bars represent the standard deviation and significance was calculated of $N = 3$ independent experiments. Statistical analysis revealed no significant changes between the represented conditions. (**C**) Activity of non-optimized high- and low-dose drug combinations in comparison to the ODCREMP characterized in Caki-1-SR clone 1 and RPTEC cells. Error bars represent the standard deviation and significance was calculated of $N = 3$ independent experiments; *** $p < 0.001$.

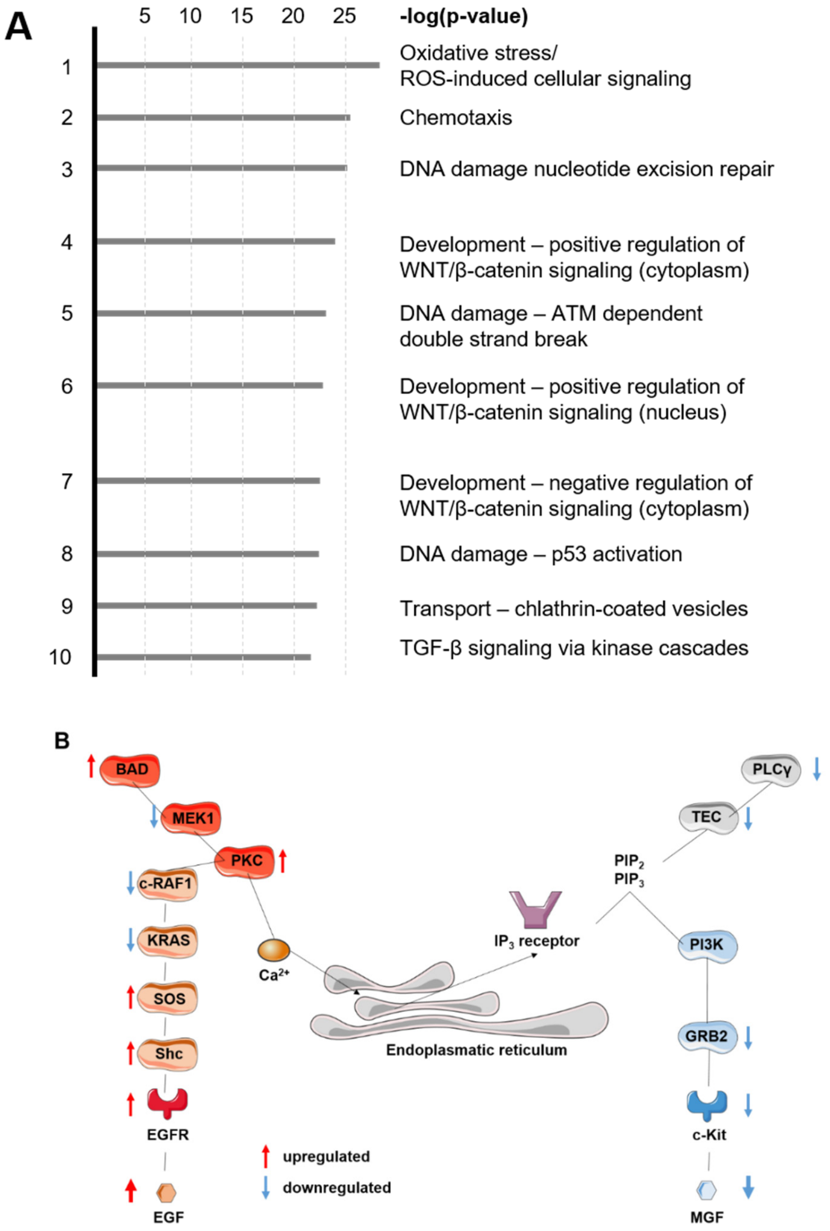

Figure S11. Global pathway analysis to extract information on the effect of the ODCREMP on cellular signaling and cellular mechanisms. (**A**) Pathway analysis performed with MetaCore™ highlighting significantly differentially regulated pathways after ODCREMP treatment that are not connected to metabolic pathways.The top ten hits in accordance to the –log(*p*-value) are listed. (**B**) Network analysis of the main signaling pathways highlighting up- and down-regulated proteins involved in the signal transduction downstream of EGFR and c-Kit mainly via the mitogen-activated kinase pathway. Legend: BAD = Bcl-2-associated death promoter; c-KIT = tyrosine-protein kinase KIT; c-RAF = serine/threonine-specific protein kinases; EGF(R) = endothelial growth factor (receptor); GRB2 = growth factor receptor-bound protein 2; KRAS = GTPase; MEK = mitogen-activated protein kinase kinase; MGF = mechano growth factor; PKC = protein kinase C; PI3K = phosphoinositide 3-kinase; PLC = phospholipase C; Shc = SHC-transforming protein 1; SOS = guanine nucleotide exchange factor (Son of Sevenless); TEC =tyrosine-protein kinase Tec.

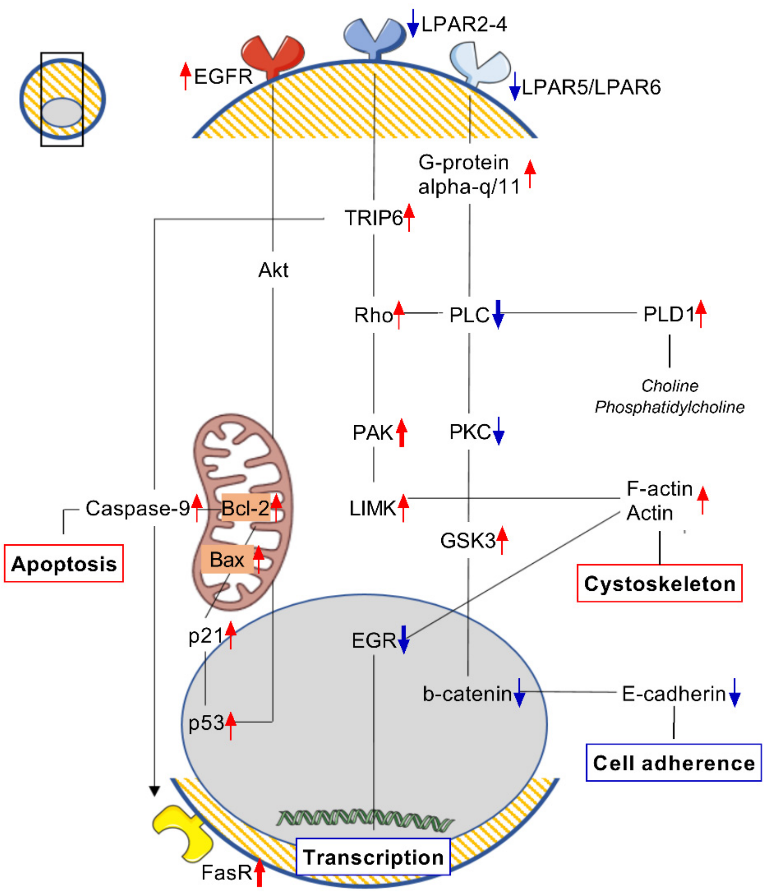

Figure S12. Pathway analysis highlighting the cellular alterations after 24 hour ODCREMP treatment. Map of the molecular pathway analysis performed with MetaCore™ demonstrating the consequences of ODCREMP treatment applied for 24 hours. As a result, proteins regulating the dynamic cytoskeleton arrangement become upregulated, which links to a decrease in cell adherence. In parallel, proteins involved in the induction of apoptosis become upregulated. Further downstream the transcription is downregulated connected to the upregulation of the cytoskeleteon arrangement. Legend: Akt = protein kinase B; Bax = Bcl-2-associated X protein; Bcl-2 = B-cell lymphoma 2; EGR = early growth response protein; FasR = apoptosis antigen 1; GSK3 = Glycogen synthase kinase 3; LIMK = serine/threonine-protein (Lim) kinase; LPAR = lysophosphatidic acid receptor; NEV = normalized expression value; PAK = serine/threonine (Pak) kinase; PKC = protein kinase C; PLC = phospholipase C; PLD1 = phospholipase D1; TRIP6 = thyroid receptor-interacting protein; red upward arrow = upregulation; blue downward arrow = downregulation.

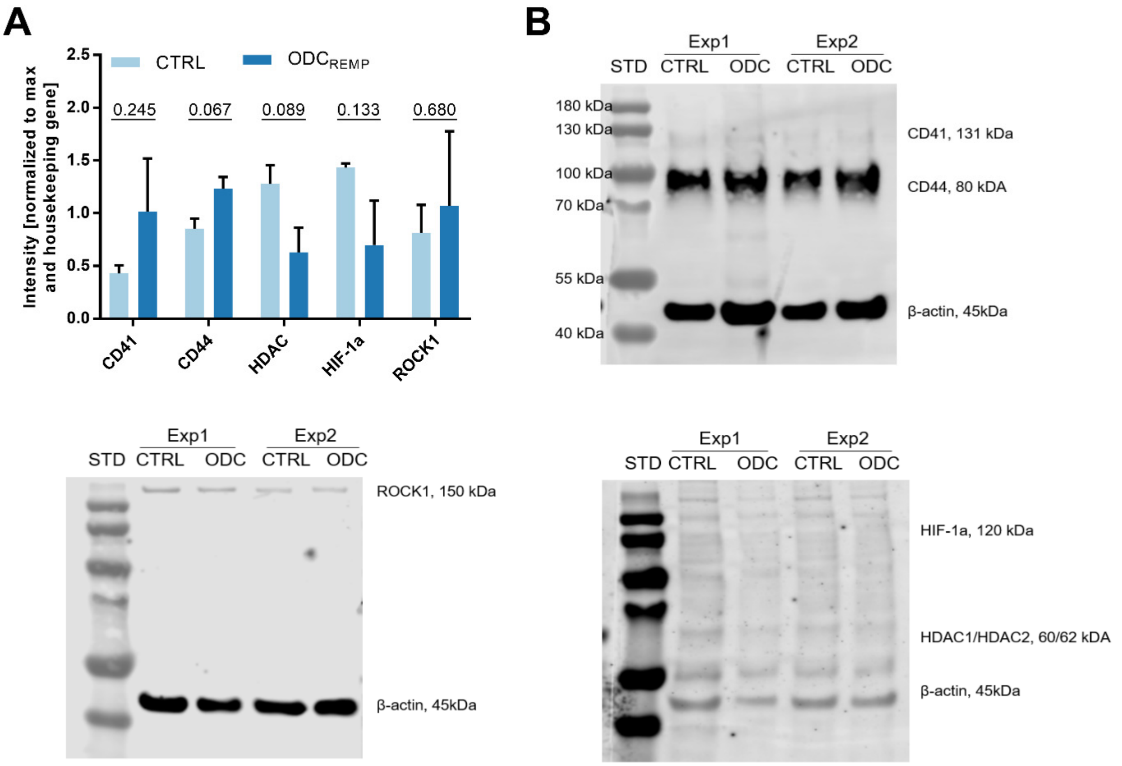

Figure S13. Protein and RNA expression of CD41, CD44, HDAC, HIF-1α and ROCK1. (**A**) Bar graphs demonstrating the protein expression in untreated and ODCREMP treated Caki-1-SR clone 1 cells of CD41 (131 kDa), CD44 (80 kDa), histone deacetylase (60/62 kDa), hypoxia inducible factor-1α (120 kDa), and Rho-associated protein kinase (150 kDa) after western blot analysis. Values above the bars represent the *p*-value calculated with unpaired t-test. All values are non-significant. (**B**) Complete western blots showing the single protein bands. Beta-actin was used as reference being a housekeeping gene. Analysis was performed for *N*=2 independent experiments (Exp1, Exp2). STD = protein ladder.

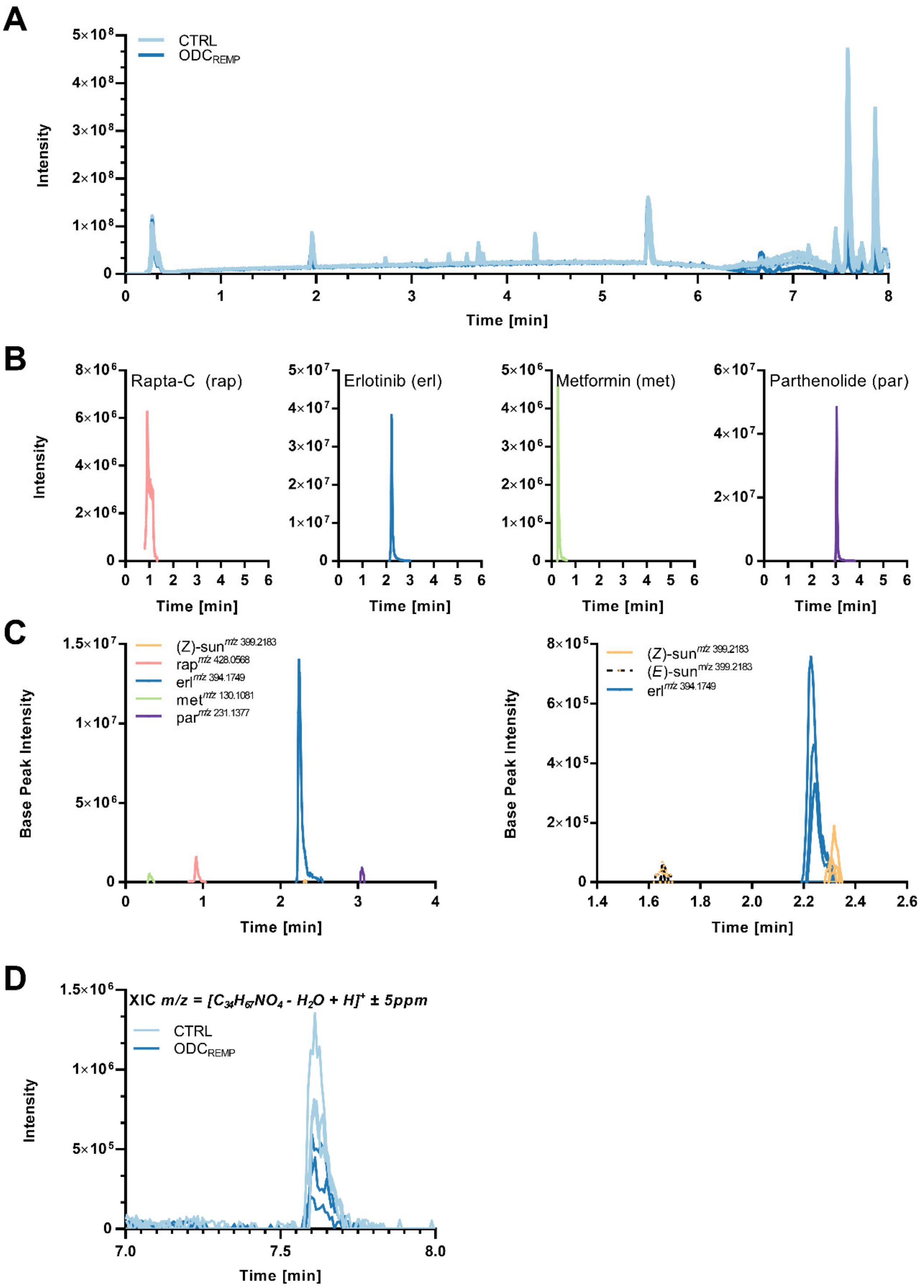

Figure S14. Positive mode LC-HRMS/MS analysis of the drugs and their metabolites in the supernatant and cell extract of Caki-1-SR clone 1 cells. (**A**) Reversed phase LC-HRMS chromatogram (positive mode) of Caki-1-SR clone 1 cells treated for 24 hours with the ODCREMP vs. control (Base Peak Intensity). (**B**) Chromatogram of standard solutions of the four drugs contained in the ODCREMP. Drugs were dissolved in DMSO and diluted in methanol to the same concentrations as applied in the combination onto the cells. (**C**) Extracted ion chromatograms of the supernatant (left graph) of Caki-1-SR clone 1 cells treated for 24 hours with the ODCREMP. All drugs were detected together with (*Z*)-stereoisomer of sunitinib [3] In the cell extract (right graph) of Caki-1-SR clone 1 cells traces of erlotinib, (*Z*)- and (*E*)-sunitinib were detected, other parent drugs could not be detected. (D) Example Extracted Ion Chromatogram of a significantly dysregulated ceramide ([Cer

14:0;O_2/20:1;O; $C_{34}H_{67}NO_4$ - H_2O +H]$^+$) in Caki-1-SR clone 1 cells treated for 24 hours with the ODCREMP versus control (CTRL).

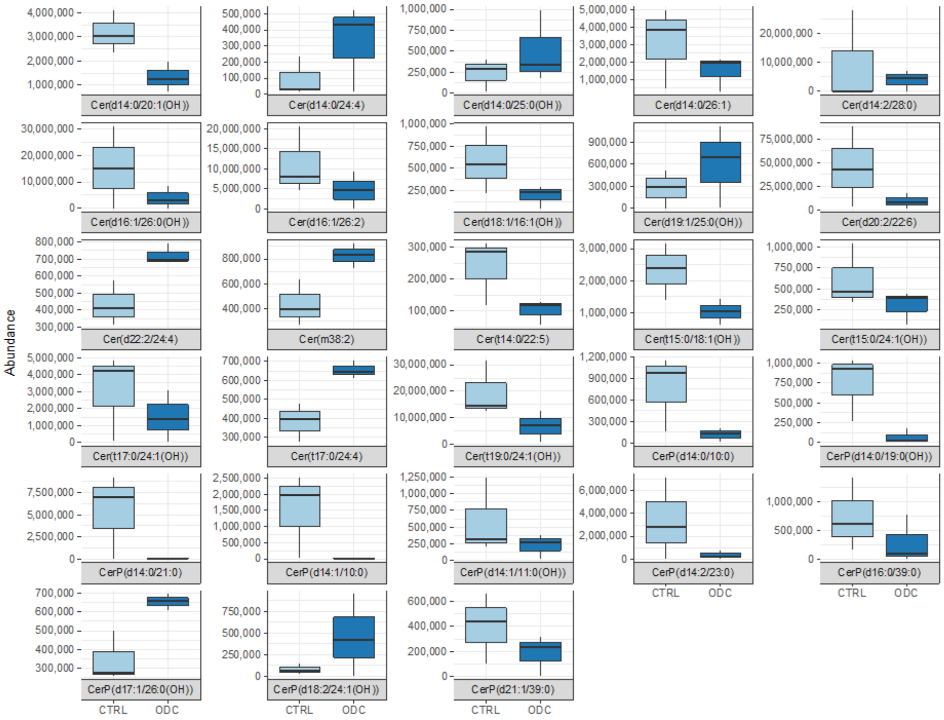

Figure S15. Ceramides after ODCREMP treatment. Normalized peak areas of 23 features annotated as ceramides significantly increased or decreased with either $p < 0.05$ or abs(log(fold change)) > 1. Features are labelled according to RefMet [4]; Metabolomics Workbench has been used to extract the common names for structural features with complete regiochemistry. The molecular product ion and neutral-loss data is diagnostic for head groups, acyl chains and other fragments. Examples are phosphatidylcholine (PC) 16:0_18:1, ceramide (Cer) 18:1;O_2/24:1; more information is provided in **Suppl Table S9**. Errors represent the standard deviation. $N = 3$.

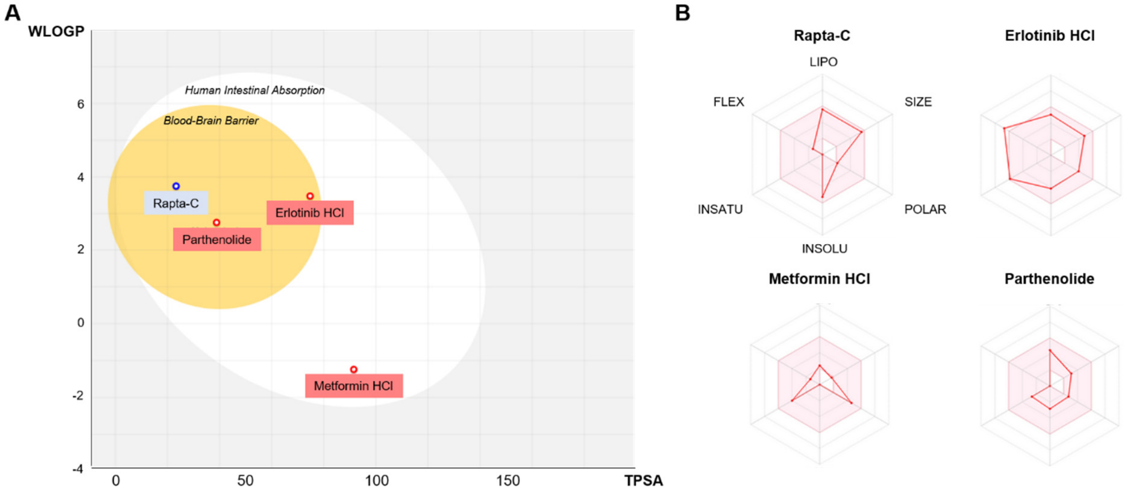

Figure S16. In silico prediction of ADME characteristics of the drugs contained in the ODCREMP. (**A**) Boiled egg graphic to present the absorption in the human intestine and the penetration of the blood brain barrier of the drugs included in the ODCREMP. (**B**) Graphical representation of the physicochemical properties, lipophilicity, pharmacokinetics, drug-likeness and medicinal chemistry for drug development (protein pattern recognition of drug) as a spider blot. Graphics were adapted from SwissADME[5]. Legend: WLOGP = model prediction (implementation of atomistic method based on fragmental system of Wildman and Crippe); TPSA = score of total charge; LIPO = lipophilicity; INSOLU = solubility; INSATU = saturation; FLEX = flexibility.

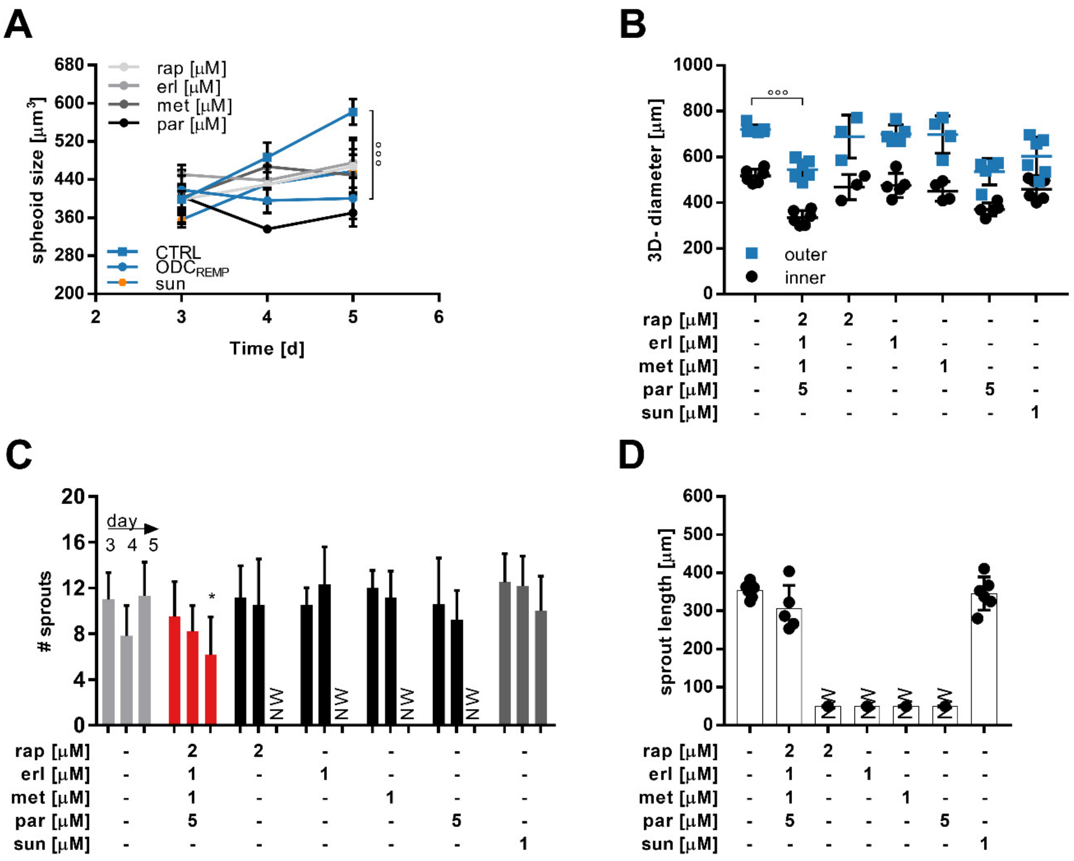

Figure S17. Analysis of the cell migration of Caki-1-SR clone 1 based 3D co-cultures in response to ODCREMP treatment. (**A**) Spheroid size of Caki-1-SR clone 1 3D co-cultureses (3Dcc) in milieu with high rigidity by supplement (0.5 mg/mL collagen type 1). (**B**) Measurement of the length of the margin of Caki-1-SR clone 1 spheroids on day 5. Error bars represent the standard deviation and significance was calculated of $N = 3$ independent experiments; *** $p < 0.001$. (**C**) Analysis of the number of sprouts from 3Dcc spheroids measured on day 4, 5, and 6. In certain conditions the increasing number of sprouts led to the formation of a network (NW) and single sprouts were not quantifiable. Error bars represent the standard deviation and significance was calculated of $N = 3$ independent experiments; * $p < 0.05$. (**D**) Bar graphs demonstrating the sprout length measured on day 5. Error bars represent the standard deviation ($N = 3$).

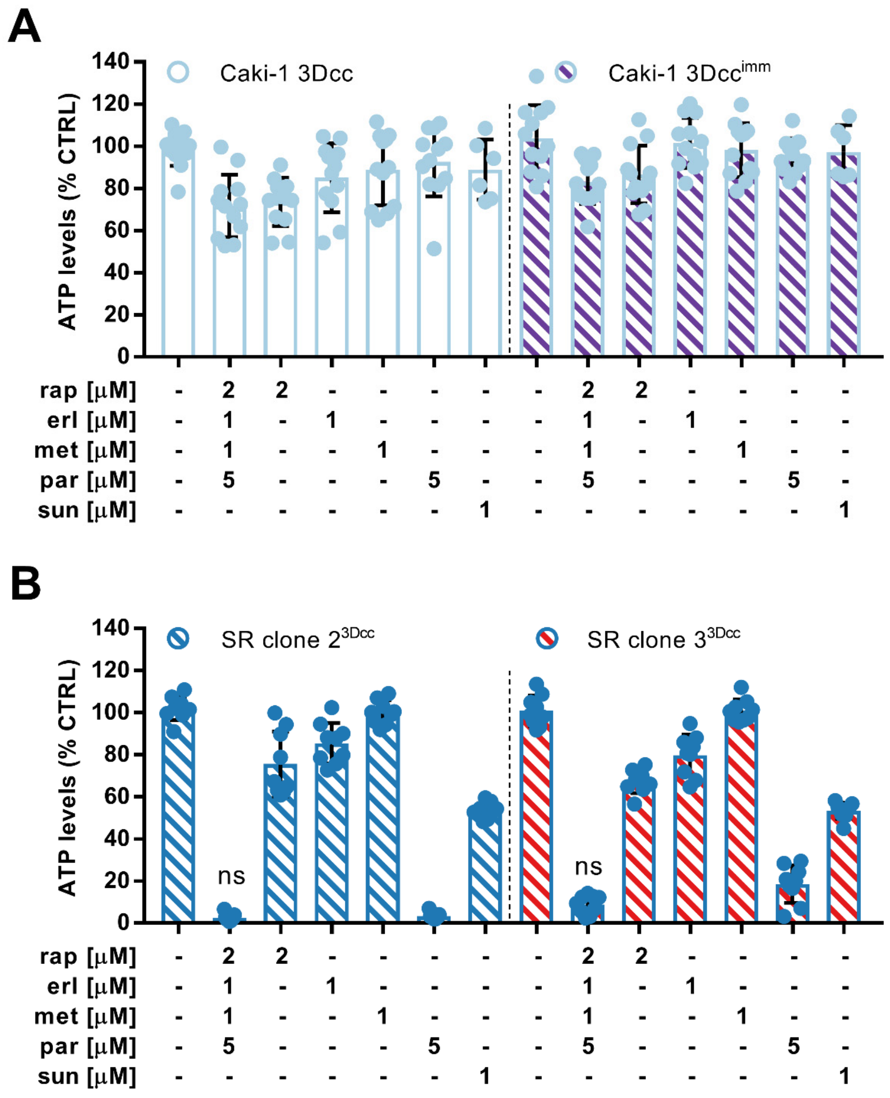

Figure S18. Adaptation of ODCREMP treatment schedule based on the readout of single 72 hour treatment and validation in sunitinib-resistant clones. (**A**) Viability, measured as ATP levels, of Caki-1 cells cultured in absence (3Dcc) and presence of immune cells (3Dcc^{imm}) in response to ODCREMP and its monotherapies together with sunitinib applied 6x in 72 hours. Error bars represent the standard deviation. (**B**) ATP levels of 3D co-cultures based on the sunitinib-resistant clones 2 and 3 after 72 hour treatment with ODCREMP and its monotherapies together with sunitinib ODCREMP and its monotherapies together with sunitinib. Error bars represent the standard deviation and significance was calculated of $N = 3$ independent experiments. Statistical analysis revealed no significant changes between the represented conditions.

Table S1. Information on selected drugs.

Drugs	MPD	Half life [h]	Dose	Side Effects **	Effect with Cisplatin
Rapta-C (rap)	-	10.39 - 12.21 [6] *	-	not reported in SIDER	-
3- Hydroxytyraminium chloride (tyr)	< 0.2 ng/L [7]	0.12	40 mg/mL	no frequency information	in combination with Rapta-T [8]
Diclofenac sodium (dic)	0.893 ± 0.51 µg/mL [9]	2 [10]	50 mg	dry skin	in combination with Rapta-T [8]

Disulfiram (dis)	380 ng/mL	60–120	250 mg	no frequency information	decrease of toxicity and increase of therapeutic index [11]
Erlotinib (erl)	0.64 μg/mL [12]	36.2 [12]	150 mg/kg	anxiety, erythema, dyspnea, headache, diarrhea, asthenia, fatigue, dermatitis, rash, dysphagia, dyspepsia, weight decreased, insomnia	combination with cisplatin inhibits growth, angiogenesis and targets erlotinib resistant cancer [13]
Famotidine (fam)	72,5 ng/mL	2.8-3.5 [14] *	20 mg	only < 10% or no frequency information	prevention and control of chemotherapy-induced gastric mucosal injury
Haloprogin (hal)	-	2.5–6	-	not reported in SIDER	in combination with Rapta-T [8]
Ivermectin (ive)	81 ng/mL [15]	12–36	12 mg (solution)	body temperature increase, diarrhea, headache	increases the efficacy of cisplatin [16]
Metformin (met)	1020 ng/mL [17]	23.4 [18]	1.5 g	diarrhea, gastrointestinal disorder, nausea, vomiting, infection, decreased appetite, abdominal distension, hypoglycemia	synergy with oxaliplatin prevents oxaliplatin-induced chronic peripheral sensory neuropathy
Parthenolide (par)	0.5 ng/mL[19]	2.87	1–5 mg	not reported in SIDER	reduces cisplatin-induced renal damage [20]
Verapamil (ver)	29.25 ng/mL [21]	2.8-6 [22]	40 mg	infection	enhanced the renal accumulation of Pt and the nephrotoxicity of cisplatin [23]
Acetylsalicylic acid (asp)	200-1323 ng/mL	3.1 [24]	50 mg	no frequency information	positive in combo with cisplatin [25]

MPD = Dose Determined in Plasma. * in mice. ** side effects extracted from SIDER 4.1 Database (http://sideeffects.embl.de). MedDRA preferred terms were chosen, and only side effects with associated frequency at least equal to “very common” (or 10%) were kept [26].

Table S2. Selected drugs and doses from empirical and TGMO search.

**Drugs**	**TGMO search**					
	**Search 1**		**Search 2**		**Search 3**	
	**D1**	**D2**	**D1**	**D2**	**D1**	**D2**
rap	2	0.7	2	0.7	2	0.7
tyr	10	0.5	1	0.5	-	-
dic	3	1	-	-	-	-
dis	5	2.5	-	-	-	-
erl	5	1	1.5	1	1.5	1
fam	20	10	-	-	-	-
hal	5	1	1	0.5	-	-
ive	3	1	0.1	0.05	-	-

met	10	1	1.5	1	1.5	1
par	3	1	5	1	5	1
ver	10	1	-	-	-	-
asp	20	10	-	-	-	-

D = dose [μM].

Table S3. Drug-Drug interactions between different drug classes.

Class 1	Class 2	Interaction	Cross-activity
Analgesics and anti-inflammatory drugs	Chemotherapy	Analgesics as adjuvant therapy for pain management, A positive effect of combining Cox-2 inhibitors and chemotherapy on overall response rate *** [27]	-
Analgesics and anti-inflammatory drugs	Efflux pump inhibitors	Efflux pump inhibitors increase susceptibility to analgesics ** [28], enhanced activation in combination ** [29]	**Verapamil**: analgesic effect *** [30],
Analgesics and anti-inflammatory drugs	Hypertension targeting agents	-	Hypertension ***
Analgesics and anti-inflammatory drugs	Hypoglycemic regimens	-	Intake of analgesics can induce hypoglycemia *** [31]
Analgesics and anti-inflammatory drugs	Wnt signaling inhibitors [32]	-	**Aspirin**: lowering β-catenin levels *, phosphorylation of β-catenin and TCF4[33], indirect Wnt signaling blockade via NFκB [34],
Chemotherapy	Efflux pump inhibitors	Efflux pump inhibitors revert resistance to chemotherapy *** [35]	**Verapamil**: increase in the efficacy of chemotherapy */** [36, 37]
Chemotherapy	Wnt signaling inhibitors	Wnt signaling inhibitors decrease resistance to chemotherapy *	-
Efflux pump inhibitors	Hypoglycemic regimens	Verapamil: decreases uptake and glucose-lowering effect of metformin *** [38]	**Verapamil**: benefit for the treatment of endogenous hyperinsulinemic hypoglycemia ***, reduction of hypoglycemic episodes after bariatric surgery ***, a decrease of hypoglycemia induced brain damage *** [39] **Metformin**: reduced risk of cardiovascular events *** Verapamil: prevention of pancreatic β cell loss */*** [40]
Efflux pump inhibitors	Wnt signaling inhibitors	Efflux pump inhibitors partially increase the sensitivity to ivermectin	**Verapamil**: suppression of β-catenin/Wnt signaling *
Hypoglycemic regimens	Wnt signaling inhibitors		**Metformin**:

targeting of Wnt/ β-catenin pathway directly * [41, 42]<br>Ivermectin:<br>glucose level alteration and cholesterol levels *

* in vitro or in vivo model, ** in parasites and bacteria, *** in human.

Table S4. Selection of 22 up- and down-regulated genes comparing ODCREMP to CTRL applied for 24 hours.

Gene	Description	FC	*p*-value with FDR	Regulation
ETV1	ETS variant transcription factor 1	-4.127	0.0361	downregulated
LYPD1	LY6/PLAUR domain containing 1	-6.136	0.0173	downregulated
ABCC3	ATP binding cassette subfamily C member 3	3.360	0.0361	upregulated
ATOH8	atonal bHLH transcription factor 8	8.371	0.0361	upregulated
BLVRB	biliverdin reductase B	2.526	0.0323	upregulated
CCDC113	coiled-coil domain containing 113	5.649	0.0439	upregulated
FAM102B	family with sequence similarity 102 member B	2.051	0.0361	upregulated
FTH1	ferritin heavy chain 1	2.888	0.0094	upregulated
FTL	ferritin light chain	4.126	0.0019	upregulated
G6PD	glucose-6-phosphate dehydrogenase	2.816	0.0321	upregulated
GPC1	glypican 1	4.075	0.0242	upregulated
HIST1H2BD	NA	3.578	0.0223	upregulated
IGFBP3	insulin like growth factor binding protein 3	4.180	0.0223	upregulated
LGI2	leucine rich repeat LGI family member 2	29.505	0.0321	upregulated
NCR3LG1	natural killer cell cytotoxicity receptor 3 ligand 1	2.379	0.0361	upregulated
NDRG1	N-myc downstream regulated 1	2.705	0.0208	upregulated
NINJ1	ninjurin 1	2.522	0.0388	upregulated
NQO1	NAD(P)H quinone dehydrogenase 1	2.911	0.0019	upregulated
RRAD	RRAD, Ras related glycolysis inhibitor and calcium channel regulator	2.891	0.0201	upregulated
SMAD6	SMAD family member 6	3.980	0.0466	upregulated
TRIM16	tripartite motif containing 16	2.948	0.0094	upregulated
TRIM16L	tripartite motif containing 16 like	4.609	0.0076	upregulated

FC = fold change; FDR = false discovery rate; NA = not applicable.

Table S5. Network analysis ODCREMP 24 h to CTRL 24 h. FC ≤ -300 and ≥ 300.

Network	GO processes	Total nodes	Pathways	*p*-value
c-Src<br>β-catenin<br>GSK3 β<br>paxillin<br>Tcf(Lef)	tube morphogenesis (75.0%; 4.766×10^{-20}), cellular response to growth factor stimulus (70.8%; 4.990×10^{-20}), regulation of cell population proliferation (91.7%; 1.268×10^{-19}), response to growth factor (70.8%; 1.496×10^{-19}), circulatory system development (79.2%; 1.533×10^{-19})	26	6	6.3×10^{-26}
c-Raf-1<br>ERK1/2<br>Shc<br>c-Kit<br>PKC-α	ERBB signaling pathway (55.0%; 4.026×10^{-22}), protein phosphorylation (85.0%; 1.919×10^{-19}), intracellular signal transduction (95.0%; 7.973×10^{-19}), epidermal growth factor receptor signaling pathway (45.0%; 1.950×10^{-18}), ERBB2 signaling pathway (40.0%; 7.540×10^{-18})	29	2	1.5×10^{-21}
ARHGAP11B<br>C17orf57<br>ZNF862	receptor guanylyl cyclase signaling pathway (10.0%; 1.035×10^{-4}), cGMP biosynthetic process (10.0%; 1.284×10^{-4}), trans-synaptic signaling, modulating synaptic transmission (10.0%; 2.545×10^{-04}),	20	0	1.3×10^{-16}

LOC440093<br>ZNF720	cGMP metabolic process (10.0%; 2.732×10^{-04}), cyclic purine nucleotide metabolic process (10.0%; 3.766×10^{-04})			
DDX60L<br>RGPD8<br>Cacng6<br>CLIC2<br>GATA-1	regulation of calcium ion transmembrane transport via high voltage-gated calcium channel (57.9%; 1.838×10^{-29}), calcium ion transmembrane transport via high voltage-gated calcium channel (47.4%; 3.247×10^{-27}), membrane depolarization during AV node cell action potential (47.4%; 2.953×10^{-26}), cardiac conduction (68.4%; 5.509×10^{-26}), AV node cell to bundle of His cell signaling (47.4%; 1.686×10^{-25})	20	0	2.1×10^{-15}
ZNF785<br>Zcchc18<br>RGPD6<br>ZNF772<br>ZNF487P	protein sulfation (11.1%; 1.972×10^{-5}), N-acetylglucosamine metabolic process (11.1%; 1.258×10^{-4}), keratan sulfate biosynthetic process (11.1%; 2.360×10^{-4}), glucosamine-containing compound metabolic process (11.1%; 3.038×10^{-4}), keratan sulfate metabolic process (11.1%; 3.408×10^{-4})	20	0	6.8×10^{-15}
ZNF114<br>FLJ40142<br>PAPAS<br>P2X6<br>ZNF311	dGDP phosphorylation (5.9%; 7.203×10^{-4}), UDP phosphorylation (5.9%; 7.203×10^{-4}), dADP phosphorylation (5.9%; 7.203×10^{-4}), TDP phosphorylation (5.9%; 7.203×10^{-4}), CMP phosphorylation (5.9%; 7.203×10^{-4})	20	0	6.8×10^{-15}
C5orf35<br>Dynein axonemal light chains<br>DCAF16<br>C6orf58<br>ZNF808	detection of chemical stimulus involved in sensory perception of taste (16.7%; 1.810×10^{-5}), sensory perception of taste (16.7%; 7.639×10^{-5}), outer dynein arm assembly (11.1%; 1.631×10^{-4}), detection of chemical stimulus involved in sensory perception (27.8%; 2.636×10^{-4}), detection of chemical stimulus (27.8%; 3.622×10^{-4})	20	0	6.8×10^{-15}
4930573I19Rik<br>NPIPL3<br>FLJ20489<br>PGBD1<br>ZNF96	G protein-coupled purinergic nucleotide receptor signaling pathway (11.8%; 9.208×10^{-5}), negative regulation of adenylate cyclase activity (11.8%; 2.099×10^{-4}), negative regulation of cyclase activity (11.8%; 2.865×10^{-4}), negative regulation of lyase activity (11.8%; 3.205×10^{-4}), purinergic nucleotide receptor signaling pathway (11.8%; 3.562×10^{-4})	20	0	6.8×10^{-15}
ZNF584<br>Galectin-13<br>ZN860<br>GLYAT<br>FAM128A	benzoyl-CoA metabolic process (5.9%; 7.203×10^{-4}), regulation of endocrine process (11.8%; 1.420×10^{-3}), detection of chemical stimulus involved in sensory perception of smell (23.5%; 1.582×10^{-3}), basophil differentiation (5.9%; 2.159×10^{-3}), eosinophil fate commitment (5.9%; 2.159×10^{-3})	20	0	6.8×10^{-15}
AOC2<br>MGC35361<br>MGAM<br>POMZP3<br>APOL2	starch metabolic process (6.7%; 6.356×10^{-4}), starch catabolic process (6.7%; 6.356×10^{-4}), detection of chemical stimulus involved in sensory perception of smell (26.7%; 9.533×10^{-4}), negative regulation of lipoprotein lipid oxidation (6.7%; 1.271×10^{-3}), negative regulation of lipoprotein oxidation (6.7%; 1.271×10^{-3})	20	0	2.8×10^{-13}

Table S6. RNA sequencing data accompanying protein expression results demonstrated in Supplementary Figure S13.

Gene	Description	Fold change	*p*-value	*p*-value with FDR
CD44	CD44 molecule (Indian blood group)	−1.057	0.808	0.943
EGLN1	egl-9 family hypoxia inducible factor 1	1.120	0.331	0.709
EGLN3	egl-9 family hypoxia inducible factor 3	−1.265	0.347	0.720

GAPLINC	gastric adenocarcinoma associated, positive CD44 regulator, long intergenic non-coding RNA	−4.326	0.008	0.145
HDAC1	histone deacetylase 1	−1.070	0.257	0.643
HDAC10	histone deacetylase 10	1.184	0.190	0.576
HDAC2	histone deacetylase 2	−1.081	0.199	0.587
HDAC3	histone deacetylase 3	−1.054	0.440	0.782
HDAC4	histone deacetylase 4	1.174	0.155	0.534
HDAC5	histone deacetylase 5	1.106	0.621	0.873
HDAC6	histone deacetylase 6	1.001	0.993	0.999
HDAC7	histone deacetylase 7	1.008	0.915	0.972
HDAC8	histone deacetylase 8	−1.061	0.360	0.730
HDAC9	histone deacetylase 9	1.063	0.805	0.942
HIF1A	hypoxia inducible factor 1 subunit alpha	1.088	0.426	0.772
HIF1AN	hypoxia inducible factor 1 subunit alpha inhibitor	−1.089	0.265	0.653
HIGD1A	HIG1 hypoxia inducible domain family member 1A	−1.185	0.049	0.334
HIGD2A	HIG1 hypoxia inducible domain family member 2A	−1.018	0.840	0.954
HILPDA	hypoxia inducible lipid droplet associated	−1.052	0.631	0.875
HYOU1	hypoxia up-regulated 1	1.096	0.285	0.671
ROCK1	Rho associated coiled-coil containing protein kinase 1	1.030	0.670	0.890
ROCK1P1	Rho associated coiled-coil containing protein kinase 1 pseudogene 1	−1.200	0.603	0.866
ROCK2	Rho associated coiled-coil containing protein kinase 2	1.004	0.966	0.988

Table S7. SMILES codes for drugs.

Drug	SMILES Code retrieved from PubChem
**Rapta-C**	Cl[Ru]1234([P]56C[N@]7C[N@@](C5)C[N@@](C6)C7)(Cl)c8([H])c(C)c1([H])c2([H])c(C(C)C)3c84[H]
**Erlotinib·HCl**	COCCOC1=C(C=C2C(=C1)C(=NC=N2)NC3=CC=CC(=C3)C#C)OCCOC.Cl
**Metformin·HCl**	CN(C)C(=N)N=C(N)N.Cl
**Parthenolide**	CC1=CCCC2(C(O2)C3C(CC1)C(=C)C(=O)O3)C

Table S8. Top 15 predicted drug targets from Swiss Target Prediction.

Drug	Target family	Target class	Target	Probability > 0.05
Rapta-C	α2a adrenergic receptor	Family A G protein-coupled receptor	ADRA2A<br>ADRA2C<br>ADRA2B	0.1016
	Purinoceptor	Ligand-gated ion channel	P2RX7	0.1016
Erlotinib HCl	Serine/threonine-protein kinase	Kinase	LRRK2	1
	Protein kinase	Kinase	HIPK4	1
	Serine/threonine-protein kinase	Kinase	SBK1	1
	Phosphatidylinositol-5-phosphate 4-kinase	Kinase	PIP4K2C	1
	Receptor protein-tyrosine kinase	Kinase	ERBB2	1
	Tyrosine-protein kinase	Kinase	ABL1	1
	Vascular endothelial	Kinase	FLT1	1

	growth factor receptor 1			
	Platelet-derived growth factor receptor beta	Kinase	PDGFRB	1
	Stem cell growth factor receptor	Kinase	KIT	1
	Vascular endothelial growth factor receptor 3	Kinase	FLT4	1
	Tyrosine-protein kinase receptor	Kinase	FLT3	1
	Platelet-derived growth factor receptor alpha	Kinase	PDGFRA	1
	Epidermal growth factor receptor	Kinase	EGFR	1
	Tyrosine-protein kinase receptor	Kinase	RET	1
	Tyrosine-protein kinase	Kinase	YES1	1
Metformin HCl	Thrombin	Protease	F2	0.0238
Parthenolide	Apoptosis regulator	Ion channel	BCL2L1	0.0536
	Cyclooxygenase	Oxidoreductase	PTGS2	0.6255
			PTGS1	0.0536
	Epoxid hydrolase	Protease	EPHX1	0.0536
	Glycine receptor	Ligand-gated ion channel	GLRA2	0.0536
	Inhibitor of NFκB	Kinase	IKBKB	0.0536
	MAP Kinase	Kinase	MAPK14	
			MAPK10	
			MAPK9	
	Nitric oxide synthase	Enzyme	NOS2	0.6255
	Protein farnesyltransferase	Enzyme	FNTA	0.1171
			FNTB	0.1171
	Cytochrome P450	Cytochrome	CYP19A1	0.0626
	Glucose transporter	Electrochemical transporter	SLC2A1	0.0536
	p53-binding protein	Nuclear protein	MDM2	0.0536
	Programmed cell death protein	unclassified	PDCD4	0.0536
	Protein kinase C	Kinase	PRKCD	0.0626
			PRKCE	0.0626
			PRKCA	0.0536
			PRKCQ	0.0536
			PRKCG	0.0536
	Proto-oncogene	Transcription factor	JUN	0.0536
		Guanine nucleotide exchange factors	VAV	0.0536
	Splicing factor	unclassified	SF3B3	0.0536

Table S9. Specification to lipids depicted in Figure 2E and Supplementary Figure S15 done with RefMet [4] hosted by Metabolomics Workbench.

Metabolite name	Super class	Main class	Sub class	Formula
13-Methylheptatriacontane	Fatty Acyls	Hydrocarbons	Hydrocarbons	$C_{38}H_{78}$
Cer 14:0; O2/20:1; O	Sphingolipids	Ceramides	Dihydroceramides	$C_{34}H_{67}NO_4$
GalCer 14:1; O2/16:0; O	Sphingolipids	Glycosphingolipids	Hexocyl ceramides	$C_{36}H_{69}NO_9$

GalCer 14:2; O2/26:2	Sphingolipids	Glycosphingolipids	Hexocyl ceramides	$C_{46}H_{83}NO_8$
PS 36:8	Glycerophos-pholipids	Glycerophosphoserines	phosphatidylserine	$C_{42}H_{66}NO_{10}P$
Cer 14:0; O2/24:4	Sphingolipids	Ceramides	Dihydroceramides	$C_{38}H_{69}NO_3$
Cer 14:0; O2/25:0; O	Sphingolipids	Ceramides	Dihydroceramides	$C_{39}H_{79}NO_4$
Cer 14:0; O2/26:1	Sphingolipids	Ceramides	Dihydroceramides	$C_{40}H_{79}NO_3$
Cer 14:0; O2/28:0	Sphingolipids	Ceramides	Dihydroceramides	$C_{42}H_{85}NO_3$
Cer 16:1; O2/26:0; O	Sphingolipids	Ceramides	Ceramides	$C_{42}H_{83}NO_4$
Cer 16:1; O2/26:2	Sphingolipids	Ceramides	Ceramides	$C_{42}H_{79}NO_3$
Cer 18:1; O2/16:1; O	Sphingolipids	Ceramides	Ceramides	$C_{34}H_{65}NO_4$
Cer 19:1; O2/25:0; O	Sphingolipids	Ceramides	Ceramides	$C_{44}H_{87}NO_4$
Cer 20:2; O2/22:6	Sphingolipids	Ceramides	Ceramides	$C_{42}H_{69}NO_3$
Cer 22:2; O2/24:4	Sphingolipids	Ceramides	Ceramides	$C_{46}H_{81}NO_3$
Cer 38:2; O	Sphingolipids	Ceramides	Ceramides	$C_{38}H_{73}NO_2$
Cer 14:0; O3/22:5	Sphingolipids	Ceramides	PhytoCer	$C_{36}H_{63}NO_4$
Cer 15:0; O3/18:1; O	Sphingolipids	Ceramides	PhytoCer	$C_{33}H_{65}NO_5$
Cer 15:0; O3/24:1; O	Sphingolipids	Ceramides	PhytoCer	$C_{39}H_{77}NO_5$
Cer 17:0; O3/24:1; O	Sphingolipids	Ceramides	PhytoCer	$C_{41}H_{81}NO_5$
Cer 17:0; O3/24:4	Sphingolipids	Ceramides	PhytoCer	$C_{41}H_{75}NO_4$
Cer 19:0; O3/24:1; O	Sphingolipids	Ceramides	PhytoCer	$C_{43}H_{85}NO_5$
CerP 14:0; O2/10:0	Sphingolipids	Ceramides	Ceramide-1-Phosphate	$C_{24}H_{50}NO_6P$
CerP 14:0; O2/19:0; O	Sphingolipids	Ceramides	Ceramide-1-Phosphate	$C_{33}H_{68}NO_7P$
CerP 14:0; O2/21:0	Sphingolipids	Ceramides	Ceramide-1-Phosphate	$C_{35}H_{72}NO_6P$

Table S10. In silico predicted pharmacokinetics using SwissADME.

Parameter	Rapta-C	Erlotinib-HCl	Metformin-HCl	Parthenolide
GI absorption	high	high	high	high
BBB permeant	yes	yes	no	yes
P-gp substrate	yes	no	no	no
CYP inhibition	no	yes	no	no

GI = gastrointestinal absorption; BBB = blood-brain barrier passage; P-gp = permeability glycoprotein; CYP = cytochromes in mitochondrial membrane (1A2 – subtypes).

Table S11. Overview of drugs and stock solution preparation.

Drug	Mw [g/mol]	Solvent	Concentration of stock solution [mg/mL]	Molarity [mol/L]
rap	461	DMSO	50	0.109
tyr	189.6	H_2O	10	0.053
dic	318.1	H_2O	10	0.031
dis	296.5	DMSO	10	0.034
erl	129.9	DMSO	15	0.116
fam	337.4	DMSO	40	0.119
hal	361.4	DMSO	10	0.028
ive	875.1	DMSO	40	0.023
met	165.6	H_2O	30	0.181
par	248.2	DMSO	20	0.077
ver	491.1	DMSO	20	0.041
asp	180	DMSO	20	0.111

Table S12. Fluorochrome-coupled antibodies for flow cytometry analysis.

Marker	Fluorochrome	Antibody Type	Dilution	Provider	Reference
CD3	PE-Cy7	Mouse a-human IgG1	1:1000	Biolegend	100220

CD4	BUV737	Mouse a-human IgG1	1:1000	BD Bioscience	557842
CD10	PE	Mouse a-human IgG1	1:500	BD Bioscience	557143
CD31	BV421	Mouse a-human IgG1	1:1000	BD Bioscience	564089
CD45	BV786	Mouse a-human	1:1000	BD Bioscience	563716
CD54 (ICAM-1)	BV711	Mouse a-human IgG3	1:1000	BD Bioscience	564078
CD274 (PD-L1)	APC	Mouse a-human IgG1	1:1000	Biolegend	329708
CD11b	BUV395	Rat a-human IgG2b	1:500	BD Bioscience	563553
CD14	APC	Mouse a-human IgG2a	1:500	Biolegend	325608
a-Fibroblast	PE-Vio770	a-human	1:500	Miltenyi Biotec	130-100-138

a = anti; Ig = immunoglobulin; ICAM = intracellular adhesion molecule; PD-L1 = programmed death-ligand 1

References

1. Thonusin, C.; IglayReger, H.B.;Soni, T.;Rothberg, A.E.;Burant, C.F.;Evans, C.R. Evaluation of intensity drift correction strategies using MetaboDrift, a normalization tool for multi-batch metabolomics data. *J. Chromatogr. A* **2017**, *1523*, 265–274, doi:https://doi.org/10.1016/j.chroma.2017.09.023.
2. Pang, Z.; Chong, J.;Li, S.;Xia, J. MetaboAnalystR 3.0: Toward an Optimized Workflow for Global Metabolomics. *Metabolites* **2020**, *10*, doi:10.3390/metabo10050186.
3. Rausch, M.;Rutz, A.;Allard, P.M.;Delucinge-Vivier, C.;Docquier, M.;Dormond, O.;Wolfender, J.L.;Nowak-Sliwinska, P. Molecular and functional analysis of sunitinib resistance induction in human renal cell carcinoma cells. *Int. J. Mol. Sci.* **2021**, 22, 6467, doi:10.3390/ijms22126467.
4. Fahy, E.; Subramaniam, S. RefMet: A reference nomenclature for metabolomics. *Nat. Methods* **2020**, *17*, 1173–1174, doi:10.1038/s41592-020-01009-y.
5. Daina, A.; Michielin, O.;Zoete, V. SwissADME: A free web tool to evaluate pharmacokinetics, drug-likeness and medicinal chemistry friendliness of small molecules. *Sci. Rep.* **2017**, *7*, 42717, doi:10.1038/srep42717.
6. Berndsen, R.H.; Weiss, A.; Abdul, U.K.;Wong, T.J.; Meraldi, P.; Griffioen, A.W.; Dyson, P.J.; Nowak-Sliwinska, P. Combination of ruthenium(II)-arene complex [Ru(eta(6)-p-cymene)Cl2(pta)] (RAPTA-C) and the epidermal growth factor receptor inhibitor erlotinib results in efficient angiostatic and antitumor activity. *Sci. Rep.* **2017**, *7*, 43005, doi:10.1038/srep43005.
7. MacGregor, D.A.; Smith, T.E.; Prielipp, R.C.;Butterworth, J.F.;James, R.L.;Scuderi, P.E. Pharmacokinetics of dopamine in healthy male subjects. *Anesthesiology* **2000**, *92*, 338–346.
8. Riedel, T.;Demaria, O.;Zava, O.;Joncic, A.;Gilliet, M.;Dyson, P.J. Drug Repurposing Approach Identifies a Synergistic Drug Combination of an Antifungal Agent and an Experimental Organometallic Drug for Melanoma Treatment. *Mol. Pharm.* **2018**, *15*, 116–126, doi:10.1021/acs.molpharmaceut.7b00764.
9. Hasan, S.M.;Ahmed, T.;Talib, N.;Hasan, F. Pharmacokinetics of diclofenac sodium in normal man. *Pak. J. Pharm. Sci.* **2005**, *18*, 18–24.
10. Altman, R.;Bosch, B.;Brune, K.;Patrignani, P.;Young, C. Advances in NSAID development: Evolution of diclofenac products using pharmaceutical technology. *Drugs* **2015**, *75*, 859–877, doi:10.1007/s40265-015-0392-z.
11. Stewart, D.J.;Verma, S.;Maroun, J.A. Phase I study of the combination of disulfiram with cisplatin. *Am. J. Clin. Oncol.* **1987**, *10*, 517–519, doi:10.1097/00000421-198712000-00012.
12. Lu, J.F.;Eppler, S.M.;Wolf, J.;Hamilton, M.;Rakhit, A.;Bruno, R.;Lum, B.L. Clinical pharmacokinetics of erlotinib in patients with solid tumors and exposure-safety relationship in patients with non-small cell lung cancer. *Clin. Pharm.* **2006**, *80*, 136–145, doi:10.1016/j.clpt.2006.04.007.
13. Lee, J.G.;Wu, R. Erlotinib-cisplatin combination inhibits growth and angiogenesis through c-MYC and HIF-1α in EGFR-mutated lung cancer in vitro and in vivo. *Neoplasia* **2015**, *17*, 190–200, doi:10.1016/j.neo.2014.12.008.
14. Chremos, A.N. Clinical pharmacology of famotidine: A summary. *J. Clin. Gastroenterol.* **1987**, *9 Suppl 2*, 7–12.
15. González Canga, A.;Sahagún Prieto, A.M.;Diez Liébana, M.J.;Fernández Martínez, N.;Sierra Vega, M.;García Vieitez, J.J. The pharmacokinetics and interactions of ivermectin in humans--a mini-review. *AAPS J.* **2008**, *10*, 42–46, doi:10.1208/s12248-007-9000-9.
16. Zhang, X.;Qin, T.;Zhu, Z.;Hong, F.;Xu, Y.;Zhang, X.;Xu, X.;Ma, A. Ivermectin Augments the In Vitro and In Vivo Efficacy of Cisplatin in Epithelial Ovarian Cancer by Suppressing Akt/mTOR Signaling. *Am. J. Med. Sci.* **2020**, *359*, 123–129, doi:10.1016/j.amjms.2019.11.001.
17. Gong, L.;Goswami, S.;Giacomini, K.M.;Altman, R.B.;Klein, T.E. Metformin pathways: Pharmacokinetics and pharmacodynamics. *Pharm. Genom.* **2012**, *22*, 820–827, doi:10.1097/FPC.0b013e3283559b22.
18. Kajbaf, F.;Bennis, Y.;Hurtel-Lemaire, A.S.;Andrejak, M.;Lalau, J.D. Unexpectedly long half-life of metformin elimination in cases of metformin accumulation. *Diabet. Med.* **2016**, *33*, 105–110, doi:10.1111/dme.12959.
19. Curry, E.A., 3rd;Murry, D.J.;Yoder, C.;Fife, K.;Armstrong, V.;Nakshatri, H.;O'Connell, M.;Sweeney, C.J. Phase I dose escalation trial of feverfew with standardized doses of parthenolide in patients with cancer. *Invest. New Drugs* **2004**, *22*, 299–305, doi:10.1023/B:DRUG.0000026256.38560.be.

20. Sohma, I.;Fujiwara, Y.;Sugita, Y.;Yoshioka, A.;Shirakawa, M.;Moon, J.H.;Takiguchi, S.;Miyata, H.;Yamasaki, M.;Mori, M.; et al. Parthenolide, an NF-kappaB inhibitor, suppresses tumor growth and enhances response to chemotherapy in gastric cancer. *Cancer Genom. Proteom.* **2011**, *8*, 39–47.
21. John, D.N.;Fort, S.;Lewis, M.J.;Luscombe, D.K. Pharmacokinetics and pharmacodynamics of verapamil following sublingual and oral administration to healthy volunteers. *Br. J. Clin. Pharmacol.* **1992**, *33*, 623–627, doi:10.1111/j.1365-2125.1992.tb04091.x.
22. Gupta, S.;Modi, N.B.;Sathyan, G.;Ho Pl, P.L.;Aarons, L. Pharmacokinetics of controlled-release verapamil in healthy volunteers and patients with hypertension or angina. *Biopharm. Drug Dispos.* **2002**, *23*, 17–31.
23. Uozumi, J.;Ueda, T.;Yasumasu, T.;Koikawa, Y.;Kumazawa, J. Calcium blockers enhance cisplatin-induced nephrotoxicity in rats. *Int. Urol. Nephrol.* **1992**, *24*, 549–553, doi:10.1007/bf02550124.
24. Nagelschmitz, J.;Blunck, M.;Kraetzschmar, J.;Ludwig, M.;Wensing, G.;Hohlfeld, T. Pharmacokinetics and pharmacodynamics of acetylsalicylic acid after intravenous and oral administration to healthy volunteers. *Clin. Pharm.* **2014**, *6*, 51 – 59, doi:10.2147/CPAA.S47895.
25. Crabb, S.J.;Martin, K.;Abab, J.;Ratcliffe, I.;Thornton, R.;Lineton, B.;Ellis, M.;Moody, R.;Stanton, L.;Galanopoulou, A.; et al. COAST (Cisplatin ototoxicity attenuated by aspirin trial): A phase II double-blind, randomised controlled trial to establish if aspirin reduces cisplatin induced hearing-loss. *European journal of cancer* **2017**, *87*, 75–83, doi:10.1016/j.ejca.2017.09.033.
26. Kuhn, M.;Letunic, I.;Jensen, L.J.;Bork, P. The SIDER database of drugs and side effects. *Nucleic Acids Res.* **2016**, *44*, D1075–1079, doi:10.1093/nar/gkv1075.
27. Dai, P.;Li, J.;Ma, X.P.;Huang, J.;Meng, J.J.;Gong, P. Efficacy and safety of COX-2 inhibitors for advanced non-small-cell lung cancer with chemotherapy: A meta-analysis. *Onco. Targets.* **2018**, *11*, 721–730, doi:10.2147/ott.S148670.
28. Laudy, A.E.;Mrowka, A.;Krajewska, J.;Tyski, S. The Influence of Efflux Pump Inhibitors on the Activity of Non-Antibiotic NSAIDS against Gram-Negative Rods. *PLoS ONE* **2016**, *11*, e0147131, doi:10.1371/journal.pone.0147131.
29. Tyers, M.;Wright, G.D. Drug combinations: A strategy to extend the life of antibiotics in the 21st century. *Nat. Rev. Microbiol.* **2019**, *17*, 141–155, doi:10.1038/s41579-018-0141-x.
30. Mosaffa, F.;Salimi, A.R.;Lahiji, F.;Kazemi, M.;Mirkheshti, A.R. Evaluation of the analgesic effect of 2 doses of verapamil with bupivacaine compared with bupivacaine alone in supraclavicular brachial plexus block. *MJIRI* **2007**, *21*, 87–90.
31. Golightly, L.K.;Simendinger, B.A.;Barber, G.R.;Stolpman, N.M.;Kick, S.D.;McDermott, M.T. Hypoglycemic effects of tramadol analgesia in hospitalized patients: A case-control study. *J. Diabetes Metab. Disord.* **2017**, *16*, 30, doi:10.1186/s40200-017-0311-9.
32. Ahmed, K.;Shaw, H.V.;Koval, A.;Katanaev, V.L. A Second WNT for Old Drugs: Drug Repositioning against WNT-Dependent Cancers. *Cancers* **2016**, *8*, doi:10.3390/cancers8070066.
33. Dihlmann, S.;Siermann, A.;von Knebel Doeberitz, M. The nonsteroidal anti-inflammatory drugs aspirin and indomethacin attenuate beta-catenin/TCF-4 signaling. *Oncogene* **2001**, *20*, 645–653, doi:10.1038/sj.onc.1204123.
34. Gala, M.K.;Chan, A.T. Molecular pathways: Aspirin and Wnt signaling-a molecularly targeted approach to cancer prevention and treatment. *Clin. Cancer Res.* **2015**, *21*, 1543–1548, doi:10.1158/1078-0432.CCR-14-0877.
35. Gottesman, M.M.;Pastan, I.H. The Role of Multidrug Resistance Efflux Pumps in Cancer: Revisiting a JNCI Publication Exploring Expression of the MDR1 (P-glycoprotein) Gene. *J. Natl. Cancer Inst.* **2015**, *107*, doi:10.1093/jnci/djv222.
36. Zhao, L.;Zhao, Y.;Schwarz, B.;Mysliwietz, J.;Hartig, R.;Camaj, P.;Bao, Q.;Jauch, K.W.;Guba, M.;Ellwart, J.W.; et al. Verapamil inhibits tumor progression of chemotherapy-resistant pancreatic cancer side population cells. *Int. J. Oncol.* **2016**, *49*, 99–110, doi:10.3892/ijo.2016.3512.
37. Liu, Y.;Lu, Z.;Fan, P.;Duan, Q.;Li, Y.;Tong, S.;Hu, B.;Lv, R.;Hu, L.;Zhuang, J. Clinical efficacy of chemotherapy combined with verapamil in metastatic colorectal patients. *Cell Biochem. Biophys* **2011**, *61*, 393–398, doi:10.1007/s12013-011-9198-0.
38. Cho, S.K.;Kim, C.O.;Park, E.S.;Chung, J.Y. Verapamil decreases the glucose-lowering effect of metformin in healthy volunteers. *Br. J. Clin. Pharm.* **2014**, *78*, 1426–1432, doi:10.1111/bcp.12476.
39. Jackson, D.A.;Michael, T.;Vieira de Abreu, A.;Agrawal, R.;Bortolato, M.;Fisher, S.J. Prevention of Severe Hypoglycemia-Induced Brain Damage and Cognitive Impairment With Verapamil. *Diabetes* **2018**, *67*, 2107–2112, doi:10.2337/db18-0008.
40. Poudel, R.R.;Kafle, N.K. Verapamil in Diabetes. *Indian J. Endocrinol. Metab.* **2017**, *21*, 788–789, doi:10.4103/ijem.IJEM_190_17.
41. Melnik, S.;Dvornikov, D.;Muller-Decker, K.;Depner, S.;Stannek, P.;Meister, M.;Warth, A.;Thomas, M.;Muley, T.;Risch, A.; et al. Cancer cell specific inhibition of Wnt/beta-catenin signaling by forced intracellular acidification. *Cell Discov.* **2018**, *4*, 37, doi:10.1038/s41421-018-0033-2.
42. Banerjee, P.;Dutta, S.;Pal, R. Dysregulation of Wnt-Signaling and a Candidate Set of miRNAs Underlie the Effect of Metformin on Neural Crest Cell Development. *Stem Cells* **2016**, *34*, 334–345, doi:10.1002/stem.2245.
